# Reconstruction of Family-Level Phylogenetic Relationships within Demospongiae (Porifera) Using Nuclear Encoded Housekeeping Genes

**DOI:** 10.1371/journal.pone.0050437

**Published:** 2013-01-23

**Authors:** Malcolm S. Hill, April L. Hill, Jose Lopez, Kevin J. Peterson, Shirley Pomponi, Maria C. Diaz, Robert W. Thacker, Maja Adamska, Nicole Boury-Esnault, Paco Cárdenas, Andia Chaves-Fonnegra, Elizabeth Danka, Bre-Onna De Laine, Dawn Formica, Eduardo Hajdu, Gisele Lobo-Hajdu, Sarah Klontz, Christine C. Morrow, Jignasa Patel, Bernard Picton, Davide Pisani, Deborah Pohlmann, Niamh E. Redmond, John Reed, Stacy Richey, Ana Riesgo, Ewelina Rubin, Zach Russell, Klaus Rützler, Erik A. Sperling, Michael di Stefano, James E. Tarver, Allen G. Collins

**Affiliations:** 1 Gottwald Science Center, University of Richmond, Richmond, Virginia, United States of America; 2 Nova Southeastern University Oceanographic Center, Dania Beach, Florida, United States of America; 3 Department of Biological Sciences, Dartmouth College, Hanover, New Hampshire, United States of America; 4 Harbor Branch Oceanographic Institute, Florida Atlantic University, Fort Pierce, Florida, United States of America; 5 Museo Marino de Margarita, Boulevard de Boca Del Rio, Boca del Rio, Nueva Esparta, Venezuela; 6 Department of Biology, University of Alabama at Birmingham, Birmingham, Alabama, United States of America; 7 Sars International Centre for Marine Molecular Biology, Thormøhlensgt, Bergen, Norway; 8 IMBE-UMR7263 CNRS, Université d'Aix-Marseille, Station marine d'Endoume, Marseille, France; 9 Department of Systematic Biology, Evolutionary Biology Centre, Uppsala University, Uppsala, Sweden; 10 Departamento de Invertebrados, Museu Nacional/Universidade Federal do Rio de Janeiro, Rio de Janeiro, Rio de Janeiro, Brazil; 11 Departamento de Genética, IBRAG, Universidade do Estado do Rio de Janeiro, Rio de Janeiro, Rio de Janeiro, Brazil; 12 Department of Invertebrate Zoology, National Museum of Natural History, Smithsonian Institution, Washington, D.C., United States of America; 13 School of Biological Sciences, MBC, Queen's University, Belfast, United Kingdom; 14 National Museums Northern Ireland, Holywood, Northern Ireland, United Kingdom; 15 School of Earth Sciences and School of Biological Sciences, The University of Bristol, Bristol, United Kingdom; 16 Museum of Comparative Zoology, Department of Organismic and Evolutionary Biology, Harvard University, Cambridge, Massachusetts, United States of America; 17 Harvard University, Department of Earth and Planetary Science, Cambridge, Massachusetts, United States of America; 18 School of Earth Sciences, University of Bristol, Bristol, United Kingdom; 19 National Systematics Laboratory of NOAA's Fisheries Service, National Museum of Natural History, Smithsonian Institution, Washington, D.C., United States of America; University of Connecticut, United States of America

## Abstract

**Background:**

Demosponges are challenging for phylogenetic systematics because of their plastic and relatively simple morphologies and many deep divergences between major clades. To improve understanding of the phylogenetic relationships within Demospongiae, we sequenced and analyzed seven nuclear housekeeping genes involved in a variety of cellular functions from a diverse group of sponges.

**Methodology/Principal Findings:**

We generated data from each of the four sponge classes (i.e., Calcarea, Demospongiae, Hexactinellida, and Homoscleromorpha), but focused on family-level relationships within demosponges. With data for 21 newly sampled families, our Maximum Likelihood and Bayesian-based approaches recovered previously phylogenetically defined taxa: *Keratosa^p^*, *Myxospongiae^p^*, *Spongillida^p^*, *Haploscleromorpha^p^* (the marine haplosclerids) and *Democlavia^p^*. We found conflicting results concerning the relationships of *Keratosa^p^* and *Myxospongiae^p^* to the remaining demosponges, but our results strongly supported a clade of *Haploscleromorpha^p^*+*Spongillida^p^*+*Democlavia^p^*. In contrast to hypotheses based on mitochondrial genome and ribosomal data, nuclear housekeeping gene data suggested that freshwater sponges (*Spongillida^p^*) are sister to *Haploscleromorpha^p^* rather than part of *Democlavia^p^*. Within *Keratosa^p^*, we found equivocal results as to the monophyly of Dictyoceratida. Within *Myxospongiae^p^*, Chondrosida and Verongida were monophyletic. A well-supported clade within *Democlavia^p^*, *Tetractinellida^p^*, composed of all sampled members of Astrophorina and Spirophorina (including the only lithistid in our analysis), was consistently revealed as the sister group to all other members of *Democlavia^p^*. Within *Tetractinellida^p^*, we did not recover monophyletic Astrophorina or Spirophorina. Our results also reaffirmed the monophyly of order Poecilosclerida (excluding Desmacellidae and Raspailiidae), and polyphyly of Hadromerida and Halichondrida.

**Conclusions/Significance:**

These results, using an independent nuclear gene set, confirmed many hypotheses based on ribosomal and/or mitochondrial genes, and they also identified clades with low statistical support or clades that conflicted with traditional morphological classification. Our results will serve as a basis for future exploration of these outstanding questions using more taxon- and gene-rich datasets.

## Introduction

Sponges belong to an ancient metazoan lineage with a fossil record that stretches back to the late Cryogenian >635 Myr ago [1]–[3]. Some estimates place their appearance at nearly 800 Myr ago [4], [5]. As a sister group (or groups) to all the other animals in the metazoan tree of life, sponges represent a fulcrum point in the history of animal life lying at the junction between single-celled ancestors and the rest of Metazoa. Sponges have also been important ecosystem engineers throughout much of their history, e.g., as major reef-builders during the Upper Devonian, Upper Permian, and through a major portion of the Jurassic [6], [7]. In modern oceans, poriferans continue to perform important ecological functions as water filterers, bioeroders, structural habitat providers, microbial symbiont incubators, dissolved organic carbon sinks, natural product biosynthesizers, chemical accumulators, and potential marine pathogen reservoirs [8]–[15]. As one of the most diverse taxa of extant sessile invertebrates [16], a detailed exploration of poriferan evolutionary relationships will yield important insights into many phases of metazoan history.

Due to their simple bodies with a paucity of easily accessible morphological traits, sponges are notoriously resistant to attempts at taxonomic classification [16]. Indeed, taxonomic controversy extends from the highest levels of classification (e.g., whether the phylum Porifera is monophyletic [17]–[20]) to whether particular genera belong to one or another family (e.g., [21]), or even whether different nominal species are truly distinct (e.g., [22], [23]). In the mid-1980s, van Soest [24] presented a call to include explicitly phylogenetic perspectives in sponge systematics through cladistic analysis. Since that time, phylogenetic classification has permeated the field of sponge taxonomy (e.g., [25]–[38]). As currently envisioned, four classes comprise the phylum Porifera: Calcarea = (Calcispongiae plus the fossil group Heteractinida), Demospongiae, Homoscleromorpha, and Hexactinellida [39]. Ample evidence exists to conclude that each of these classes is monophyletic, and so each has been provided with an explicit phylogenetic definition [40]. Indeed, substantial evidence is accumulating for the existence of various sponge clades at different levels [40], [41], and throughout this paper, we will differentiate between Linnean taxa and those clades that have been provided with explicitly phylogenetic definitions by italicizing phylogenetically defined taxa and following them with a superscript p, as in *Demospongiae^p^* (i.e., *PhyloCode* designations).

A major challenge to scientists working in this field has been the identification of appropriate markers for addressing the daunting task of dealing with ancient divergences among the diverse assortment of poriferan taxa. Evolutionary relationships across the most diverse class of Porifera, Demospongiae, have mainly been addressed with three sets of phylogenetic markers: ribosomal DNA sequences [17], [42], complete mitochondrial genome sequences [43], and amino acid sequences that code for seven nuclear housekeeping genes [18], [44]. A broad correspondence in inferences about demosponge phylogeny exists between these three sets of data (see discussion below), but both of the latter two sets of data have been sampled from a far more limited number of taxa. The Porifera Tree of Life project (www.portol.org) employs a variety of tools to integrate morphological and molecular data and to expand the diversity of sponge taxa used to elucidate all levels of sponge phylogeny. In this study, we report findings based on a significant expansion (38 new samples from 38 species representing 30 families, including 21 families newly sampled) of the nuclear housekeeping gene dataset first developed for metazoan-wide phylogenetic and molecular dating analyses [45], [46] and later applied by Sperling et al. [18], [44] to sponges, with a thorough taxonomic vetting process and a slightly modified phylogenetic analysis focused on relationships within *Demospongiae^p^*.

## Results

Extraction of high quality RNA for subsequent cDNA synthesis and cloning was a significant hurdle, curtailing use of some samples (e.g., lithistids), even though a large number of archived specimens were available for potential study [47]. Several hundred cDNAs were cloned and sequenced, but only 159 usable sequences were generated due to the amplification of non-sponge contaminants (Tables 1–2). We evaluated single gene phylogenies (ALD, ATPB, etc.) including all the members of each gene family that could be identified in GenBank (via reciprocal blasting) to identify and remove potential paralogs. Our dataset for phylogenetic analysis contains 2,033 amino acid characters and a total of 68 sponge species representing 48 of 137 accepted and recently proposed families of Porifera [38], [40], [48], including 51 species from 37 of 91 families recognized for Demospongiae (Table 1). The most appropriate models of amino acid evolution, as determined by ProtTest [49] for the various datasets (i.e., all genes, each individual gene, etc.), nearly always involved some variant of the LG matrix [50] (Table 3). Maximum likelihood mapping, performed for each gene under the best fitting model, among those implemented in Treepuzzle [51], showed that each of the seven considered genes convey enough phylogenetic signal to be considered potentially useful phylogenetic markers to resolve the relationships within Demospongiae (Figs. S1, S2, S3, S4, S5, S6, S7). Bayesian cross-validation [52] analyses showed that the CAT based models (CAT and CAT-GTR) fit our dataset significantly better than any empirical site-homogeneous time reversible model tested (WAG+G, and LG+G). Cross-validation also showed that the CAT-based models fit the data better than the more complex site-homogeneous time reversible model: the mechanistic amino acid-GTR (Table 4) model. Accordingly, hypothesized relationships obtained with homogeneous time-reversible models (e.g. LG or GTR), where differing from those obtained in our CAT and particularly CAT-GTR analysis, could be considered inferior. That said, just five of the resolved nodes in the Bayesian analysis contradict those in the ML-based topology and none of these have pp values>0.90.

**Table 1:** Annotated list of samples and sequences used for analysis. New sequences and samples are indicated in bold.

Higher Clades/Classification and Identification		Voucher #	ALD	ATPB	CAT	EF1a	MAT	PFK	TPI	PorToL ID
***Keratosa^p^*** **, Dendroceratida**										
Dictyodendrillidae	*Igernella notabilis* (1)	USNM_1148204	GQ332402	GQ330912	GQ336998	GQ330927	GQ330916	GQ330918	GQ330922	NA
Dictyodendrillidae	***Igernella notabilis***	**USNM_1133861**	**JQ606746**	**JQ606789**		**JQ606696**	**JQ680966**		**JQ680967**	**P153**
***Keratosa^p^*** **, Dictyoceratida**										
Dysideidae	*Dysidea etheria* (2)	USNM_1148214	GQ332403	GQ330913	GQ336999	GQ330928		GQ330919		NA
Irciniidae	***Ircinia strobilina***	**USNM_1153592**			**JQ680968**	**JQ606699**		**JQ606661**		**TOL24**
Irciniidae	*Ircinia strobilina*	USNM_1148130	GQ331021	GQ330993	GQ331006	GQ330979		GQ330952	GQ330939	NA
Spongiidae	***Hippospongia lachne***	**USNM_1154092**		**JQ606797**	**JQ606729**	**JQ606706**				**RWT1816**
Thorectidae	***Hyrtios proteus***	**USNM_1133719**	**JQ606755**	**JQ606799**	**JQ606731**	**JQ606708**		**JQ606668**	**JQ606775**	**P14**
***Myxospongiae^p^*** **, Chondrosida**										
Chondrillidae	*Chondrilla caribensis* (3)	USNM_1148122	GQ332401	HM859880	GQ336997	GQ330926	GQ330915		HM859889	NA
Halisarcidae	*Halisarca* sp.	USNM_1148131	GQ331020	GQ330992		HM859888	GQ330965		GQ330938	NA
***Myxospongiae^p^*** **, Verongida**										
Aplysinidae	***Aiolochroia crassa***	**USNM_1133710**	**JQ606737**		**JQ606713**	**JQ606687**				**P4**
Aplysinidae	***Aplysina fistularis***	**USNM_1153593**	**JQ606736**	**JQ606781**	**JQ606712**	**JQ606685**	**JQ606671**			**TOL 25**
Aplysinidae	*Aplysina fulva*	USNM_1148123	GQ331013	GQ330987	GQ331000	GQ330973	GQ330958		GQ330932	NA
Aplysinidae	*Verongula rigida*	NA	GQ331026	HM859882	GQ331012		GQ330971		GQ330946	NA
***Spongillida^p^***										
Spongillidae	*Trochospongilla pennsylvanica*	NA	DQ087496		DQ087498	DQ087497	DQ087499		DQ087500	NA
Spongillidae	*Ephydatia fluviatilis* (4)	NA	AY580188	AY580189	AY580190	AY580191	AY580192	AY580193	AB000891	NA
***Haploscleromorpha^p^***										
Callyspongiidae	***Callyspongia vaginalis***	**USNM_1154088**		**JQ606785**	**JQ606716**	**JQ606690**	**JQ606672**	**JQ606656**	**JQ606760**	**RWT1812**
Chalinidae	***Haliclona manglaris***	**USNM_1133711**	**JQ606741**		**JQ606717**	**JQ606691**		**JQ606655**	**JQ606761**	**P5**
Chalinidae	*Haliclona (Haliclona)* sp.	NA	GQ331014	GQ330988	GQ331001	GQ330974	GQ330959	GQ330949	GQ330933	NA
Chalinidae	*Haliclona* sp.	NA	GQ331019	GQ330991	GQ331005	GQ330978	GQ330964		GQ330937	NA
Niphatidae	***Amphimedon compressa***	**USNM_1153590**	**JQ606749**	**JQ606793**	**JQ680969**	**JQ606701**	**JQ606679**		**JQ606768**	**TOL20**
Niphatidae	*Amphimedon queenslandica*	NA	(16)	(16)	(16)	(16)	(16)	(16)		NA
Petrosiidae	***Petrosia ficiformis***	**MCZ_DNA105722**		KA659909	KA659907	KA659906	KA659904	KA659905	KA659901	
Petrosiidae	***Xestospongia muta***	**USNM_1154090**	**JQ606750**					**JQ606663**	**JQ606771**	**RWT1813**
Phloeodictyidae	***Aka coralliphaga***	**USNM_1133740**	**JQ606751**	**JQ606795**	**JQ606726**					**P34**
***Democlavia^p^*** **, ** ***Tetractinellida^p^*** **, Astrophorina**										
Ancorinidae	***Dercitus (Halinastra) luteus***	**USNM_1175047**		**JQ606794**	**JQ606725**	**JQ606703**	**JQ606677**		**JQ606770**	**JR190**
Geodiidae	***Geostelletta^p^ fibrosa*** (5)	**USNM_1133730**	**JQ606735**	**JQ606779**				**JQ606652**	**JQ606757**	**P24**
Geodiidae	*Geodia tumulosa* (6)	NA		GQ330990	GQ331004	GQ330977	GQ330963		GQ330936	NA
*incertae sedis*	***Characella*** ** aff. ** ***connectens*** (7)	**USNM_1175067**				**JQ606702**			**JQ606769**	**JR15**
***Democlavia^p^*** **, ** ***Tetractinellida^p^*** **, Spirophorina**										
Scleritodermidae	***Microscleroderma*** ** sp. nov.** (8)	**USNM_1133739**		**JQ606784**		**JQ606689**				**P33**
Tetillidae	*Cinachyrella apion* (9)	USNM_1153585	GQ331015	HM859881	HM859884	HM859886	GQ330960		GQ330934	NA
***Democlavia^p^*** **, Agelasida**										
Agelasidae	***Agelas conifera***	**USNM_1154089**	**JQ606734**	**JQ606778**	**JQ606711**	**JQ606684**		**JQ606651**	**JQ606756**	**RWT1814**
Hymerhabdiidae	***Cymbaxinella^p^ corrugata*** (10)	**USNM_1153725**	**JQ606739**	**JQ606782**	**JQ606714**			**JQ606653**	**JQ606758**	**TOL29**
***Democlavia^p^*** **, Axinellida**										
Raspailiidae	***Ectyoplasia ferox*** (11)	**USNM_1133718**	**JQ606753**				**JQ606680**	**JQ606666**		**P13**
***Democlavia^p^*** **, Hadromerida**										
Clionaidae	***Cliona varians***	**USNM_1154091**	**JQ606742**	**JQ606786**	**JQ606718**	**JQ606692**	**JQ606674**	**JQ606657**	**JQ606762**	**RWT1815**
Placospongiidae	***Placospongia intermedia***	**USNM_1133726**	**JQ606752**		**JQ606727**	**JQ606704**	**JQ606679**	**JQ606664**	**JQ606772**	**P20**
Polymastiidae	***Polymastia tenax***	**USNM_1133747**		**JQ606796**	**JQ606728**	**JQ606705**		**JQ606665**	**JQ606773**	**P40**
Spirastrellidae	*Spirastrella* sp. (12)	USNM_1148132	GQ331017	GQ330989	GQ331003	GQ330976	GQ330962	GQ330950	GQ330935	NA
Suberitidae	*Suberites* sp.	USNM_1148202	GQ331024	GQ330997		GQ330984	GQ330969		GQ330944	NA
Tethyidae	*Tethya californiana* (13)	USNM_1148128	GQ331025	GQ330998	GQ331011	GQ330985	GQ330970	GQ330956	GQ330945	NA
***Democlavia^p^, incertae sedis***										
Desmacellidae	***Biemna caribea***	**USNM_1175046**	**JQ606745**		**JQ606721**	**JQ606695**		**JQ606660**		**TOL27**
Desmacellidae	***Desmacella pumilio***	**USNM_1175045**	**JQ606738**	**JQ606780**		**JQ606686**	**JQ606673**		**JQ606763**	**JR19**
Dictyonellidae	**Dictyonellidae sp. nov.**	**USNM_1133716**	**JQ606740**	**JQ606783**	**JQ606715**	**JQ606688**		**JQ606654**	**JQ606759**	**P11**
Halichondriidae	***Halichondria melanadocia***	**USNM_1133755**	**JQ606747**	**JQ606790**	**JQ606722**	**JQ606697**			**JQ606765**	**P48**
Halichondriidae	*Halichondria* sp.		GQ332404	GQ330914	GQ337000	GQ330929		GQ330920	GQ330924	NA
***Democlavia^p^*** **, Poecilosclerida**										
Coelosphaeridae	***Lissodendoryx colombiensis***	**USNM_1133712**	**JQ606743**	**JQ606787**	**JQ606719**	**JQ606693**		**JQ606658**		**P6**
Crambeidae	*Monanchora arbuscula* (14)	USNM_1148203	GQ331023	GQ330996	GQ331010	GQ330983		GQ330955	GQ330943	NA
Crambeidae	***Monanchora arbuscula***	**USNM_1153736**	**JQ606744**	**JQ606788**	**JQ606720**	**JQ606694**	**JQ606675**	**JQ606659**	**JQ606764**	**TOL23**
Hymedesmiidae	***Phorbas sp. nov.***	**USNM_1133787**		**JQ606791**		**JQ606698**	**JQ606676**		**JQ606766**	**P80**
Microcionidae	*Clathria (Clathria) prolifera* (15)	USNM_1148129	DQ087472	DQ087473	DQ087474	DQ087476	DQ087477		DQ087478	NA
Mycalidae	***Mycale laevis***	**USNM_1133707**	**JQ606748**	**JQ606792**	**JQ606723**	**JQ606700**	**JQ606678**	**JQ606662**	**JQ606767**	**P1**
Tedaniidae	***Tedania ignis***	**USNM_1153591**	**JQ606754**	**JQ606798**	**JQ606730**	**JQ606707**	**JQ606681**	**JQ606667**	**JQ606774**	**TOL21**
***Calcispongiae^p^*** **, ** ***Calcaronea^p^***										
Amphoriscidae	*Leucilla nuttingi*	NA		GQ330994		GQ330980	GQ330966	GQ330953	GQ330940	NA
Leucosoleniidae	*Leucosolenia* sp.	USNM_1126268	DQ087465	DQ087466	DQ087467	DQ087468	DQ087469	DQ087470	DQ087471	NA
Leucosoleniidae	***Leucosolenia complicata***	pending	pending	pending	pending	pending	pending	pending	pending	NA
Sycettidae	*Sycon lingua*	USNM_1148127	DQ087458	DQ087459	DQ087460	DQ087461	DQ087462			NA
Sycettidae	***Sycon coactum***	**MCZ_DNA105723**	KA659914		KA659917		KA659915	ÊKA659916	ÊKA659911	NA
Sycettidae	***Sycon ciliatum***	**ZMBN_87981-2**	pending	pending	pending	pending	pending	pending	pending	NA
***Calcispongiae^p^*** **, ** ***Calcinea^p^***										
Clathrinidae	*Clathrina cerebrum*	NA	GQ331016			GQ330975	GQ330961			NA
Leucettidae	*Leucetta chagosensis*	NA	(17)	(17)		(17)	(17)			NA
***Homoscleromorpha^p^***										
Oscarellidae	*Oscarella carmela*	NA	GQ332405		GQ337001	ACL97976	GQ330917	GQ330921	GQ330925	NA
Plakinidae	***Corticium candelabrum***	MCZ_ DNA105720			KA659897		KA659898	KA659899	KA659900	NA
Plakinidae	*Plakortis angulospiculatus*	USNM_1148206	GQ331022		GQ331008	GQ330981	GQ330967		GQ330941	NA
***Hexactinellida^p^*** **, ** ***Hexasterophora^p^***										
Aphrocallistidae	*Aphrocallistes vastus*	NA		GQ330986	GQ330999	GQ330972	GQ330957	GQ330947	GQ330931	NA
Aphrocallistidae	*Heterochone calyx*	NA	(18)	(18)	(18)	(18)	(18)			
Euplectellidae	***Hertwigia falcifera***	**USNM_1175049**		**JQ606800**	**JQ606733**		**JQ606682**	**JQ606669**	**JQ606776**	**JR14**
Rossellidae	*Acanthascus dawsoni*	NA		GQ330995	GQ331009			GQ330954	GQ330942	NA
Rossellidae	***Nodastrella asconemaoida***	**USNM_1175065**		**JQ606801**		**JQ606709**	**JQ606683**		**JQ606777**	**JR11**
Rossellidae	***Bathydorus*** ** sp.**	**USNM_1175050**		**JQ606802**	**JQ606732**	**JQ606710**		**JQ606670**		**JR09**
**Non-Sponge Metazoans**										
Cnidaria	*Nematostella vectensis*	NA	(16)	(16)	(16)	(16)	(16)	(16)	(16)	NA
Cnidaria	*Metridium senile*	NA	AAT06124	AAT06144		AAT06185	AAT06205	AAT06226	AAT06245	NA
Cnidaria	*Acropora millepora*	NA	(18)	(18)	(18)	(18)	(18)		(18)	NA
Placozoa	*Trichoplax adhaerens*	NA	(18)	(18)	(18)	(18)	(18)	(18)	(18)	NA

(1) Formerly identified as Darwinella muelleri (Darwinellidae) in Sperling et al. (2007); specimen from the Gulf of Mexico.

(2) Formerly identified as Dysidea camera in Sperling et al. (2007), and as Dysidea sp. in GenBank.

(3) Formerly identified as Chondrilla sp. in Sperling et al. (2007) and as Chondrilla nucula in GenBank.

(4) Formerly labeled as Clypeatula cooperensis in Sperling et al. (2004) and Ephydatia cooperensis in GenBank, but synonomized with Ephydatia fluviatilis in WPD.

(5) Presently in WPD as Stelleta fibrosa as part of family Ancorinidae, but see Cárdenas et al. (2011) for updated classification.

(6) Formerly identified as Geodia gibberosa in Sperling et al. (2009) and in GenBank; G. tumulosa was resurected by Cárdenas et al. (2011).

(7) Characella presently classified in the WPD within Pachastrellidae, but is incertae sedis according to Cárdenas et al. (2011).

(8) Microscleroderma and its family Scleritodermidae presently classified in the WPD within Lithistida, well-known as a polyphyletic group, but is transferred to Spirophorida by Cárdenas et al. (2012).

(9) Formerly identified as Cinachyrella alloclada in Sperling et al. (2009) and in GenBank.

(10) Presently in WPD as Axinella corrugata as part of family Axinellidae within Halichondrida, but see Gazave et al. (2010) and Morrow et al. (2012), who updated its classification.

(11) Ectyoplasia and Raspailiidae presently classified in the WPD within Poecilosclerida, but was transferred to Axinellida by Morrow et al. (2012).

(12) Formerly identified as Damiria sp. in Sperling et al. (2009) and in GenBank.

(13) Formerly identified as Tethya aurantia in Sperling et al. (2009), and as Tethya actinia in GenBank.

(14) Formerly identified as Spirastrella coccinea in Sperling et al. (2009) and in GenBank.

(15) Formerly labeled as Microciona prolifera in Peterson & Butterfield (2005) and in GenBank, and as Clathria (Microciona) prolifera in Sperling et al. (2009).

(16) Derived from genomic traces, as reported in Sperling et al. (2007).

(17) Derived from genomic traces, as reported in Sperling et al. (2010).

(18) Derived from genomic traces, as reported in Sperling et al. (2009).

**Table 2:** Summary of genes and taxa for analysis* by poriferan clade.

	ALD	ATPB	CAT	EF1A	MAT	PFK	TPI	NHK7	NHK6	NHK5	NHK4
*Keratosa^p^*	5	6	6	7	2	5	4	6	6	7	7
*Myxospongiae^p^*	6	5	5	5	4	0	4	6	6	6	6
*Haploscleromorpha^p^*	7	7	8	7	5	6	7	9	9	9	9
*Spongillida^p^*	2	1	2	2	2	1	2	2	2	2	2
*Tetractinellida^p^*	2	5	3	5	3	1	5	5	5	6	6
Other *Democlavia^p^*	19	18	17	19	12	15	18	21	21	21	21
***Demospongiae^p^***	41	42	41	45	30	28	40	49	49	51	51
*Calcispongiae^p^*	7	6	5	7	8	5	5	8	8	8	8
*Hexactinellida^p^*	2	6	5	4	4	4	4	6	6	6	6
*Homoscleromorpha^p^*	2	2	2	2	3	2	3	3	3	3	3
**TOTAL**	54	56	53	58	43	39	52	66	66	68	68

* NHK7 refers to the complete dataset, while NHK6-4 refer to datasets where the markers CAT, EF1A, and ATPB are successively removed.

**Table 3:** Amino acid model selection, used for maximum likelihood searches on different datasets*.

Dataset	Most Appropriate Model	Criterion	Model Assumed
NHK7	LG+G+I+F	all AIC	LG+G+F
ALD	LG+G	AICc-1,2	LG+G
ATPB	WAG+G+I	all AIC	WAG+G
CAT	LG+G+I	AIC, AICc-1,3	LG+G
EF1A	LG+G+I+F	AIC, AICc-1,3	LG+G+F
MAT	LG+G+I	AICc-1,2	LG+G
PFK	LG+G	all AIC	LG+G
TPI	LG+G+I	all AIC	LG+G
NHK6	LG+G+I	all AIC	LG+G
NHK5	LG+G+I	all AIC	LG+G
NHK4	LG+G+I	AICc-1,2	LG+G

* NHK7 refers to the complete dataset, while NHK6-4 refer to datasets where the markers CAT, EF1A, and ATPB are successively removed.

**Table 4:** Model cross validation performed using CAT-GTR as the reference model.

Models Compared	Mean Score	Standard Deviation	
CAT+gamma	CAT-GTR+gamma	−66.0556*	27.2128
GTR+gamma	CAT-GTR+gamma	−203.2*	26.4986
LG+gamma	CAT-GTR+gamma	−201.862*	26.7209
WAG+gamma	CAT-GTR+gamma	−226.778*	32.4408

* A negative cross validation score indicates that the reference model (CAT-GTR) fits the data better then the tested model. This table indicates that CAT-GTR provides the best fit to the data (as the standard deviations around the means are not sufficient to define a confidence intervals including positive values).

**Figure 1:**
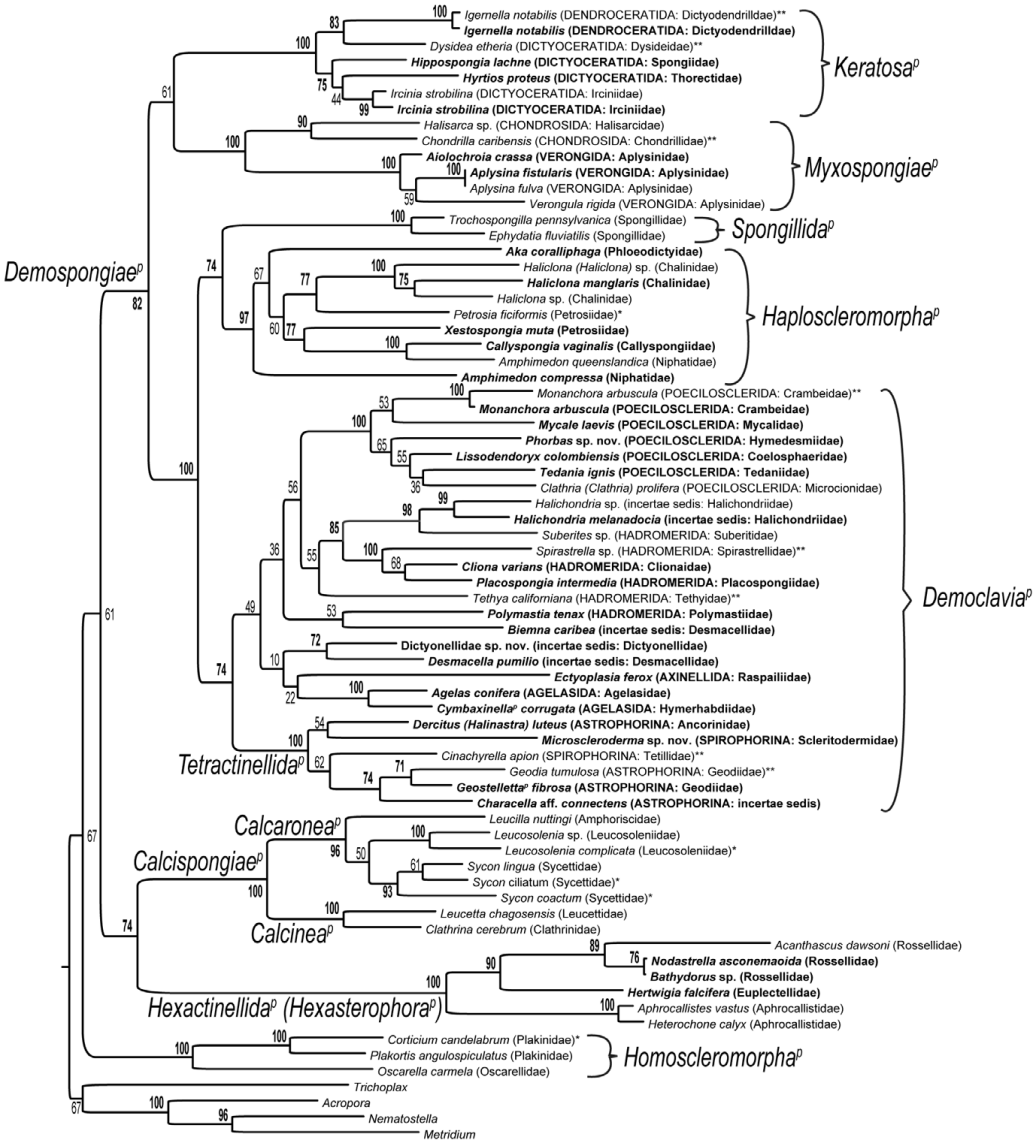
Hypothesis of demosponge relationships based on maximum likelihood analysis of seven nuclear housekeeping genes. Topology rooted on three cnidarians and the placozoan *Trichoplax*. Bootstrap indices (400 replicates) are shown at each node, with those exceeding 70 in bold. New taxa added as part of the PorToL project are indicated in bold; new taxa added from EST/genomics projects are indicated with a single asterisk; and taxa with new identifications after examination of the voucher specimen are marked with two asterisks. Clade names in italics followed by a superscript p have been phylogenetically defined in other studies (see text).

**Figure 2:**
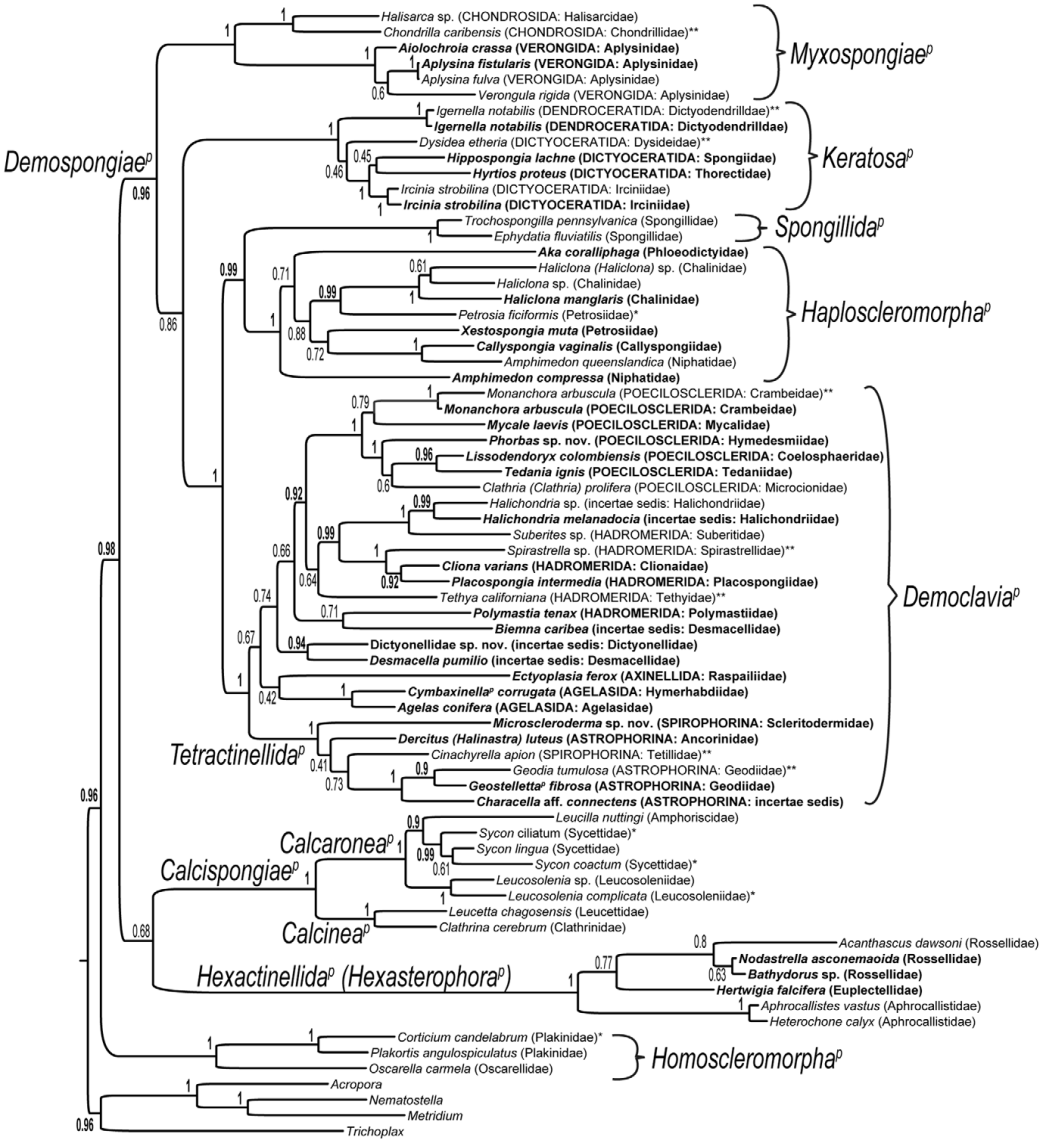
Hypothesis of demosponge relationships based on Bayesian analysis of seven nuclear housekeeping genes. Topology rooted on three cnidarians and the placozoan *Trichoplax*. Posterior probabilities are shown at each node, with those exceeding 0.90 in bold. New taxa added as part of the PorToL project are indicated in bold; new taxa added from EST/genomics projects are indicated with a single asterisk; and taxa with new identifications after examination of the voucher specimen are marked with two asterisks. Clade names in italics followed by a superscript p have been phylogenetically defined in other studies (see text).

The partitioned ML analysis of the combined data had the same topology as that found when assuming a single model of amino acid evolution (LG+F+G). Additionally, no major differences were found when comparing a Bayesian analysis performed under LG+G, the ML analysis performed using LG+F+G, and the ML analysis performed using multiple partitions. We used this topology as the reference point for comparing the different analyses (Fig. 1). The Bayesian topology (Fig. 2) is highly consistent with the ML-based topology (Table 5). Each of the single-gene ML topologies (Figs. S8, S9, S10, S11, S12, S13, S14) differs from that derived from the combined dataset. An ordered ranking of how well the single-gene topologies match our overall hypothesis, based on nodal difference is: PFK, TPI, ALD, MAT, ATPB, CAT and EF1A (Table 5). This performance is also reflected in a tabulation of whether notable clades were recovered in the single-gene topologies (Table 6), where ATPB, CAT and EF1A recovers less than half of a set of reference clades in the topology based on the combined data. ML analyses serially excluding CAT, EF1A, and ATPB resulted in topologies (Figs. S15, S16, S17) that are highly consistent with the tree based on the analysis of combined data (Table 5–6). A supertree analysis was performed to evaluate the extent to which the principal signal [53] in the single-gene partitions differed from the signal in the gene concatenation and the results showed a substantial level of agreement (Fig. S18).

**Table 5:** Nodal differences between reference topology (ML assuming LG+G+F) and topologies derived from different datasets* and analyses.

Dataset/Analysis	Percentage of Taxa in Common	Nodal Difference	Random Difference	Standard Deviation
ALD	76.4%	2.50	4.49	0.36
ATPB	83.3%	3.42	4.65	0.34
CAT	77.8%	3.48	4.55	0.34
EF1A	86.1%	3.73	4.60	0.37
MAT	65.3%	2.67	4.38	0.34
PFK	58.3%	1.91	4.15	0.34
TPI	77.8%	2.13	4.42	0.29
NHK6	100.0%	1.45	4.80	0.35
NHK5	97.2%	1.42	4.73	0.31
NHK4	97.2%	1.42	4.75	0.38
NHK7/Bayesian	100.0%	1.26	4.77	0.35

* NHK7 refers to the complete dataset, while NHK6-4 refer to datasets where the markers CAT, EF1A, and ATPB are successively removed.

**Table 6:** Comparison of clades found in NHK7* ML topology with those revealed in single-gene and other analyses.*

Clades of Interest	ML ALD	ML ATPB	ML CAT	ML EF1A	ML MAT	ML PFK	ML TPI	ML NHK6	M NHK5	ML NHK4	Bayes NHK7
Cnidaria	yes	no	yes	no	yes	yes	no	yes	yes	yes	yes
*Calcispongiae^p^*	yes	yes	yes	yes	yes	yes	yes	yes	yes	yes	yes
*Homoscleromorpha^p^*	yes	no	No	yes	yes	yes	yes	yes	yes	yes	yes
*Hexactinellida^p^*	yes	yes	yes	yes	yes	yes	yes	yes	yes	yes	yes
*Demospongiae^p^*	yes	no	no	no	no	no	no	yes	yes	yes	yes
*Keratosa^p^* (G1)	yes	yes	yes	no	yes	yes	yes	yes	yes	yes	yes
*Myxospongiae^p^* (G2)	yes	no	yes	no	yes	–	no	yes	yes	yes	yes
G1+G2	yes	no	no	no	no	–	no	yes	yes	yes	no
*Spongillida^p^*	yes	–	yes	yes	yes	–	yes	yes	yes	yes	yes
*Haploscleromorpha^p^* (G3)	yes	yes	no	yes	yes	no	yes	yes	yes	yes	yes
*Spongillida^p^*+G3	no	no	no	no	no	yes	no	yes	yes	yes	yes
*Democlavia^p^* (G4)	no	no	no	no	no	no	yes	yes	yes	yes	yes
*Tetractinellida^p^*	yes	yes	no	no	yes	–	yes	yes	yes	yes	yes
G3+G4+*Spongillida^p^*	yes	no	no	no	yes	yes	no	yes	yes	yes	yes
Clades	12/14	5/13	5/14	5/14	10/14	7/10	8/14	14/14	14/14	14/14	13/14
Percent	86%	38%	36%	36%	71%	70%	57%	100%	100%	100%	93%

* NHK7 refers to the complete dataset, while NHK6-4 refer to datasets where the markers CAT, EF1A, and ATPB are successively removed.

Nodal support for the ML-based phylogenetic hypothesis (Fig. 1) varies widely; 46 of 70 nodes have bootstrap support (bs) exceeding 70%. Similarly, although generally higher in magnitude, posterior probability (pp) values in the Bayesian topology are not universally high, with 44 of 70 nodes having values exceeding 0.90 (Fig. 2).

To test whether some of our results could be attributed to tree reconstruction artifacts we performed a variety of analyses. We first built trees using differently fitting models (WAG, LG, GTR, CAT, and CAT-GTR) and compared their results. This analysis indicated an important area of disagreement with reference to the relationships between *Keratosa^p^* and *Myxospongiae^p^* (see discussion). We performed a posterior predictive analysis to identify compositionally heterogeneous taxa. This analysis indicated that many taxa in the dataset are, indeed, compositionally heterogeneous (Table S1). The 6-categories Dayhoff recoding strategy is commonly used to ease compositional heterogeneity. We recoded our dataset using the 6-categories Dayhoff strategy and performed a posterior predictive analysis and found that the Dayhoff recoding eliminated almost all heterogeneity from the data (Table S2). CAT-GTR analyses of the Dayhoff recoded dataset found a tree (Fig. S19) that is highly comparable with the CAT-GTR tree of Fig. 2 (non-recoded data). However, results of the Bayesian analysis using Dayhoff recoded data and assuming GTR (Fig. S20) contains a key difference. In the Dayhoff recoded GTR analysis *Myxospongiae^p^* is not the sister group of *Keratosa^p^* but the sister group of all the other Demospongiae (albeit with a low PP). Analyses performed after excluding compositionally heterogeneous species, fast-evolving sites, or the outgroups consistently reiterate the results of our Bayesian analysis (compare Fig. 2 with Figs. S21, S22, S23).

## Discussion

### Sponge Classes

Analyses of the seven nuclear housekeeping gene dataset provide strong support for each of the four major clades of sponges assigned the rank of class (Calcarea, Demospongiae, Hexactinellida, and Homoscleromorpha). Because we did not include non-metazoan outgroups our results cannot be used to assess sponge monophyly. Concerning the relationships among the four sponge classes, support is generally poor. Our tree does not recover *Silicea^p^* (*Demospongiae^p^*+*Hexactinellida^p^*), which has been supported in a great deal of other works based on disparate datasets [4], [18], [19], [28], [54], but instead places *Calcispongiae^p^* with *Hexactinellida^p^* (Figs. 1–2), most likely erroneously with low support (bs = 74%; pp = 0.68). Relationships within *Calcispongiae^p^* and *Hexactinellida^p^* are consistent with previous analyses [54]–[56]. As designed, our analyses do not provide any basis for inferring relationships among the sponge classes (as they do not include non-metazoan outgroups), but rather elucidate phylogenetic relationships within *Demospongiae^p^* (Figs. 1–2).

### Major Demosponge Clades

Hypotheses derived from our analyses of nuclear housekeeping gene data (Figs. 1–2) are fairly consistent with the so-called “G clades” originally derived from analysis of ribosomal DNA data [17], and largely recovered by mitochondrial genome [43] and nuclear housekeeping gene data [18]. G1 and G2 correspond to *Keratosa^p^* and *Myxospongiae^p^*, respectively, following the names of Borchiellini et al. [17]. One key difference between the results of these studies concerns the placement of the clade containing all freshwater sponges, *Spongillida^p^*, phylogenetically defined in Cárdenas et al. [40]. Traditionally, these sponges were classified as the suborder Spongillina within the order Haplosclerida. However, ribosomal DNA and mitochondrial genome data suggested that *Spongillida^p^* falls as the earliest diverging lineage of the “G4” clade. Sperling et al. [18] found a similar clade, for which they provided a phylogenetic definition and the name *Democlavia^p^* ( = subclass Heteroscleromorpha of Cárdenas et al. [40]), with the exception that *Spongillida^p^* was found as the sister group of the marine haplosclerids. The marine haplosclerid taxa have consistently been shown to be a well-supported clade that has recently been phylogenetically defined and named *Haploscleromorpha^p^* [40].

This study finds strong support at nearly all deep nodes within *Demospongiae^p^* (Figs. 1–2), even with our more diverse taxon sampling. The clear distinction of these clades indicates that the divergence among these groups is likely ancient [4]. Thus, future genomic exploration within *Demospongiae^p^* will be guided by these emerging phylogenetic results so as to make best use of the comparative method. To be especially useful for rank-based taxonomy and nomenclature, type species within genera and type genera within families (e.g., our sampling of *Spongia officinalis*, *Halisarca dujardini*, and *Desmacella pumilio*) should be targeted whenever possible. Also, to the extent possible, type species should be collected from their respective type localities for maximum taxonomic and nomenclatural utility. For phylogenetic nomenclature, ‘specifiers’ (i.e., species, specimens or apomorphies used in *PhyloCode* definitions) should be targeted. Of course, when species are used as specifiers (which has so far usually been the case for poriferan names), their name-bearing type specimens are *de facto* specifiers (*PhyloCode*, Note 13.2.2.).

Nuclear housekeeping gene data strongly support an as yet unnamed clade containing the groups of demosponges with silica-mineralized skeletons: *Democlavia^p^*, *Haploscleromorpha^p^*, and *Spongillida^p^* (Figs. 1–2), in accordance with other analyses of ribosomal genes [17], complete mitochondrial genomes [43], and a smaller dataset of nuclear housekeeping genes [18]. Our ML and Bayesian analyses provide equivocal results concerning the phylogenetic relationships of *Keratosa^p^* and *Myxospongiae^p^*. A sister group relationship between *Keratosa^p^* and *Myxospongiae^p^* has been suggested, with only modest support, based on analyses of 18S rRNA genes [17] and complete mitochondrial genomes [43] but has also been contradicted by earlier Bayesian analyses of nuclear housekeeping genes [4], [18], [44]. Our ML topology (Fig. 1) shows *Keratosa^p^* and *Myxospongiae^p^* [which both lack mineralized skeletons (with the exception of siliceous microscleres in *Chondrilla* within *Myxospongiae^p^*: Chondrosida)] as a clade that is sister to the mineralized sponges. In contrast, the Bayesian analysis (Fig. 2) identifies *Myxospongiae^p^* as the earliest diverging clade of *Demospongiae^p^*, and shows *Keratosa^p^* as the sister group to the mineralized groups. It is important to note, however, that all site-homogeneous models (LG and GTR) display the *Keratosa^p^*+*Myxospongiae^p^* clade, while the site-heterogeneous CAT and CAT-GTR models (which fit the data better) support *Myxospongiae^p^* as the sister group of all the other demosponges. Thus, model selection is responsible for this disagreement. Because the best fitting models suggest *Myxospongiae^p^* is sister to the remaining demosponges, the contradicting results obtained using LG, GTR and WAG (*Keratosa^p^*+*Myxospongiae^p^*) are likely artifactual.

### *Keratosa^p^*

This clade is composed of members of the demosponge orders Dictyoceratida and Dendroceratida. Our sampling includes members of five of the six families: Dysideidae, Irciniidae, Spongiidae and Thorectidae in the former, Dictyodendrillidae in the latter. Ribosomal data [17] indicate that Dendroceratida is monophyletic, but our results rely on a single genus (*Igernella*) so we cannot support or refute that result. The nuclear housekeeping gene data also fail to provide support for the monophyly of Dictyoceratida, a result that has also been obtained through the analysis of ribosomal data [35], [57]. We have conflicting results concerning Dictyoceratida, with our ML-topology (Fig. 1) suggesting that dendroceratids are derived from within a paraphyletic Dictyoceratida and the Bayesian tree having a poorly supported monophyletic Dictyoceratida. The key taxon, from the perspective of this analysis, is the representative of Dysideidae. All the other dictyoceratids in our study, representing Irciniidae, Spongiidae, and Thorectidae, always form a well-supported clade. It is interesting to note that when the worst performing markers (CAT, EF1A, and ATPB) are sequentially removed from analysis, Dictyoceratida, including our representative of Dysideidae, forms a monophyletic group with strong support (Figs. S15, S16, S17).

### *Myxospongiae^p^*

Members of the orders Chondrosida and Verongida make up *Myxospongiae^p^*. Our sampling includes both families of Chondrosida (Chondrillidae and Halisarcidae), the latter of which was previously placed in its own order Halisarcida (e.g., [58]). Within Verongida, just one of the four families of Verongida (i.e., Aplysinidae) is sampled. With the present taxon sampling, our analyses support monophyly of Chondrosida, a result not obtained by some analyses of ribosomal data [17], [59], but found in others [35], [60]. However, our analysis lacks a representative of *Chondrosia*, which has proven to be a difficult taxon in relation to the question of Chondrosida monophyly [17], [59]. Similarly lacking a representative of the problematic *Chondrosia*, an analysis of complete mitochondrial genome data also supports a monophyletic *Chondrosida^p^* [43], which has nevertheless recently been given a phylogenetic definition [40]. Within Verongida, nuclear housekeeping genes support monophyly of Aplysinidae, for which we were able to sample each of its component genera (Figs. 1–2). Relationships among the three aplysinid genera (*Verongula*, *Aplysina*, and *Aiolochroia*), however, are not well supported. Based on ribosomal data, Erwin and Thacker [61] found that Aplysinidae is not monophyletic because *Verongula* grouped with members of Pseudoceratinidae and members of *Aiolochroia* grouped with Ianthellidae and Aplysinellidae. The absence of pseudoceratinids, ianthellids and aplysinellids from our samples prevents our analyses from testing these hypotheses, but if Erwin and Thacker's [61] findings are true, they would suggest that our sampling represents a more disparate group of Verongida (*Aplysina* in Aplysinidae and *Verongula* in Pseudoceratinidae) than is suggested by current taxonomy (*Aplysina* and *Verongula* in Aplysinidae). Indeed, this phylogenetic result (i.e., that *Aplysina* and *Verongula* belong to distinct families) was recently verified with mitochondrial and nuclear markers by Erpenbeck et al. [59].

### *Haploscleromorpha^p^* & *Spongillida^p^*

From a broad perspective, one of the most important outstanding questions in demosponge phylogenetics is the phylogenetic placement of the freshwater sponges, *Spongillida^p^*, which is phylogenetically defined in Cárdenas et al. [40]. Traditional taxonomy based on morphology [62] and earlier analyses of nuclear housekeeping genes [18] suggest a close relationship between *Spongillida^p^* and the marine haplosclerids, *Haploscleromorpha^p^*. In contrast, both mitochondrial genome and ribosomal data suggest that *Spongillida^p^* is sister to the rest of the *Democlavia^p^* [17], [35], [43], [63]. The results here, for the most part, agree with the former hypothesis and specifically indicate that *Spongillida^p^* is the earliest diverging lineage of the traditional order Haplosclerida (with high support, Figs. 1–2). An exception to this result is one of the single gene analyses (ALD, Fig. S8), which found *Spongillida^p^* branching among democlaviid taxa, albeit with no support. Limited taxon sampling, and in particular, the fact that our analyses do not include any representatives of the democlaviid family Scopalinidae (which was recently suggested by Morrow et al. [38] to have a close relationship to the freshwater sponges), could explain these contradicting results. In any event, it is fairly clear that *Spongillida^p^* is a distinct lineage from the marine haplosclerids.

Our sampling within *Haploscleromorpha^p^* represents five of the six accepted families. Monophyletic haplosclerid suborders Petrosina and Haplosclerina were not recovered (although support values are somewhat low at some of the deeper branches of the clade), corroborating the results of McCormack et al. [64] and Redmond et al. [35], [37]. Not surprisingly, given that studies with denser taxon sampling have shown widespread polyphyly of subtaxa within this group [35], [37], [65], we find both Petrosiidae and Niphatidae to be polyphyletic. Even at the generic level, *Amphimedon* (Niphatidae) is revealed to be polyphyletic. *Amphimedon queenslandica*, whose genome has been sequenced [66], clusters with *Callyspongia vaginalis* (Callyspongiidae) with high support, suggesting that the taxonomy of this important model organism remains confused, corroborating evidence from ribosomal data [35], [37].

### *Democlavia^p^*

*Democlavia^p^* is the most species-rich (roughly 75% of demosponge species; [38]) and diverse of the major demosponge clades, and includes the traditional orders Agelasida, Astrophorida, Hadromerida, Halichondrida, Poecilosclerida, and Spirophorida [48], several of which are already thought to not be monophyletic (as discussed below). As such, the systematics of *Democlavia^p^* presents many challenges, but important breakthroughs are being made in understanding the phylogeny of this clade based on increasingly taxon-rich analyses of ribosomal RNA and mitochondrial CO1 data [38]. Our nuclear housekeeping gene dataset and analyses provide an opportunity to test hypotheses arising from these alternative sets of data and suggest new hypotheses where previous results have provided no resolution.

Our analyses reveal a well-supported clade containing members of Astrophorina and Spirophorina (suborder designations for these taxa, following [40]), including our only sampled lithistid (*Microscleroderma* sp. nov.). Other analyses of ribosomal and mitochondrial data have revealed the same clade [17], [35], [42], [67]–[69], the phylogenetically defined *Tetractinellida^p^* [17], [40]. Although modest in support, our analyses always suggest that T*etractinellida^p^* is sister to the remaining members of *Democlavia^p^*. Our sampling of sub-order Astrophorina includes two of the six families, Ancorinidae (*Dercitus*, recently transferred from Pachastrellidae by Cárdenas et al. [70]) and Geodiidae (*Geodia tumulosa* and *Geostelletta^p^ fibrosa*), as well as an *incertae sedis* taxon, *Characella* aff. *connectens*, which was also formerly assigned to family Pachastrellidae. The latter three species form a well-supported clade, but no specific position for our representative of Ancorinidae within *Tetractinellida^p^* is supported (Figs. 1–2). The family Pachastrellidae *sensu* Maldonado [71] is based on a plesiomorphic character (streptasters; [70]) so it is no surprise that our results confirm that *Characella* and *Dercitus* do not have an especially close relationship.

Our analyses include two representatives of Spirophorina – *Cinachyrella* sp., representing the family Tetillidae, and the lithistid *Microscleroderma* sp. nov., representing the family Scleritodermidae – but there is no support for the group being monophyletic. The lithistids are a taxonomically rich group sharing a common growth form (skeleton of interlocked desmas), with 13 recognized families. Lithistids have always presented taxonomic challenges from morphological perspectives (see 72) and the redistribution of its members to different sponge clades has been proposed for quite some time [72], [73] and continues [40]. In this vein, the lithistid family Desmanthidae appears to be closely related to Dictyonellidae [38]. The presence of sigmaspires in Scleritodermidae [72] is consistent with this group being reallocated to Spirophorina within *Tetractinellida^p^* [40].

Another well-supported alliance of taxa includes most members of order Poecilosclerida that we have sampled, specifically representatives of Coelosphaeridae, Crambeidae, Hymedesmiidae, Microcionidae, Mycalidae, and Tedaniidae (Figs. 1–2). Monophyly of Poecilosclerida has been found in several analyses of ribosomal data [17], [35], [42], [74], but more recent studies with greater taxon sampling have shown the group to be polyphyletic [38], [75], as found here. Morrow et al. [38] demonstrated that the families Desmacellidae and Raspailiidae should be removed from Poecilosclerida. Our results support this action, as our representatives of these families branch deeper within *Democlavia^p^* (Figs. 1–2). Unfortunately, these data do not provide strong support for relationships within this poecilosclerid group, which remains the most species-rich order and therefore one of the more challenging clades within *Demospongiae^p^*.

The sister group to Poecilosclerida (*sensu* 38) consists of most of our sampled hadromerids as well as the family Halichondriidae from the order Halichondrida. A similar relationship was derived in Morrow et al. [38]. Within this clade, three hadromerids, *Cliona* (Clionaidae), *Placospongia* (Placospongiidae), and *Spirastrella* (Spirastrellidae) form a well-supported clade. In turn, this clade is revealed to have a relatively well-supported relationship with the families Halichondriidae and Suberitidae. The latter two families, currently classified within Halichondrida and Hadromerida, respectively, have long been known to have a close relationship [27]. Interestingly, the hadromerid *Tethya* (Tethyidae) consistently branches with this alliance of Suberitidae, Halichondriidae, and the hadromerids (representing Clionaidae, Placospongiidae and Spirastrellidae) albeit with limited support. One other hadromerid in our analysis, *Polymastia tenax*, falls outside this clade, a peculiar result given that Polymastiidae is considered among the “core” components of Hadromerida [76]. In the 28S-based analysis of Morrow et al. [38], Polymastiidae emerged as a distinct clade, sister to Suberitidae plus Halichondriidae but with low support, whereas their analysis of CO1 data recovered a clade with Polymastiidae sister to the hadromerid families Tethyidae, Hemiasterellidae, and Clionaidae, but again with only low support.

The monophyly of *Agelasida^p^* is well supported. This result is obtained only after taking into account recent findings made by Gazave et al. [36], who provided a phylogenetic definition of the clade, and corroborated by Morrow et al. [38]. In light of polyphyly of *Axinella* (order Axinellida), Gazave et al. [36] erected the taxon *Cymbaxinella^p^* for those species, including *Axinella corrugata* sampled here, with a close relationship to *Agelas* (family Agelasidae). With broader taxon sampling, Morrow et al. [38] established the new family Hymerhabdiidae for this same clade within *Agelasida^p^*. In contrast with this study [38], however, nuclear housekeeping gene data do not provide further support for a sister group relationship between *Agelasida^p^* and the clade containing the core poecilosclerids, hadromerids and Halichondriidae. The only representative of order Axinellida in our analysis is *Ectyoplasia*; the species belongs to the family Raspailiidae, which was moved from Poecilosclerida to Axinellida by Morrow et al. [38]. That study [38] also found that representatives of Desmacellidae fell in two groups, a finding we also recovered given that *Desmacella* and *Biemna* did not exhibit a particularly close relationship. It is important to note that our analysis includes the type species of *Desmacella*. Nuclear housekeeping gene data provide modest support for a relationship between *Desmacella* and the family Dictyonellidae (Figs. 1–2).

## Conclusions

As with any phylogenetic analysis, the hypotheses presented here do not represent the final statement on demosponge phylogeny. In particular, the aforementioned gaps in taxonomic sampling limit the extent to which these analyses are able to assess interesting and relevant hypotheses of demosponge relationships. Nonetheless, this analysis makes several important strides forward. First, our results bolster previous claims of the efficacy of the nuclear housekeeping gene marker set [44], albeit at a high cost in effort. Analyses of these data with enhanced taxon sampling confirm numerous phylogenetic hypotheses derived from ribosomal DNA and mitochondrial markers. Most importantly, this boosts overall confidence in the emerging picture of demosponge systematics and phylogenetics that has largely been based on ribosomal and mitochondrial markers, which are more readily obtained from sponge samples. Nevertheless, there are still key points of difference, for example the position of the freshwater *Spongillida^p^* clade, that remain to be tested by new datasets, and numerous open questions not yet satisfactorily answered by any phylogenetic analyses, such as the position of *Tetractinellida^p^* within *Democlavia^p^*, and the relationships among *Keratosa^p^*, *Myxospongiae^p^*, and the clade consisting of *Democlavia^p^*, *Haploscleromorpha^p^*, and *Spongillida^p^*. A final important advance of this study is that incorporates a diverse set of sponge systematicists engaged in transforming the taxonomy (both *PhyloCode*-based and more traditional approaches) used to describe demosponge diversity. As a new understanding of demosponge relationships emerges, the names – and possibly the rules by which we erect and use them – must change [38]–[41].

## Materials and Methods

### Ethics Statement

In accordance with policy and legal requirements associated with specimens vouchered in the collections of the Smithsonian US National Museum of Natural History (NMNH), Harbor Branch Oceanographic Institute (HBOI), Harvard Museum of Comparative Zoology (MCZ), and Zoological Museum Bergen Norway (ZMBN), all collections involved in this study were obtained with all appropriate and relevant permits. Specifically, samples from Panama were collected under a Marine Collecting Permit provided by The Republic of Panama; samples from the State of Florida were collected under a Florida recreational resident saltwater fishing license issued from Florida Fish and Wildlife Conservation Commission; and one sample from Honduras was collected with the permission of Rosa del Carmen Garcia, Directora General de Pesca y Acuicultura. No permits were required to collect sponge specimens in US territorial waters outside state boundaries, the Catalan coast of Spain, Vancouver Island, Canada, or Norway.

### Sample and sequence collection

Samples were collected from a variety of locations and stored as described below or obtained from frozen collections at the Harbor Branch Oceanographic Institute-Florida Atlantic University (Table 1; http://PorToL.org/NHK7data). To obtain RNA of sufficient quality and quantity, when possible, fresh material was collected and preserved via one of several methods. One involved placing fresh material in cold 75% ethanol with liquid changes occurring after 15 min, 1 hour and 4 hours. When available, material was also placed in RNAlater (Invitrogen), directly in TRIzol® (Invitrogen) reagent, following the manufacturer's instructions, or in liquid nitrogen. In most cases, the tissue placed directly in TRIzol® or frozen in liquid nitrogen yielded the highest quality and/or quantity of RNA. However, the most practical storage method in the field was 75% ethanol preservation and in most cases this was suitable for RNA extraction and subsequent polymerase chain reaction (PCR) amplifications from cDNA.

Following Sperling et al. [18], [44] total RNA was isolated using a one-step TRIzol® method (Invitrogen), and cDNA was synthesized from 1–2 µg RNA using RETROSCRIPT® (Ambion) reverse transcriptase using both random decamers and oligo dT primers, which were then pooled. PCR was used to amplify 7 nuclear-encoded genes: aldolase (ALD), ATP synthase beta chain (ATPB), catalase (CAT), elongation factor 1-alpha (EF1alpha), methionine adenosyltransferase (MAT), phosphofructokinase (PFK), and triose-phosphate isomerase (TPI). All primer sequences for initial PCR of housekeeping genes can be found in Sperling et al. [44]. In many cases, however, it was necessary to use nested PCR primers if amplification and re-amplification of housekeeping gene products was not possible. Table S3 provides primer sequences for nested amplifications of individual housekeeping genes. Primary or nested amplification products were cloned into PCR cloning vectors (pGEM®-T, Promega or TOPO TA®, Invitrogen) and individual clones were prepared for DNA sequencing using standard protocols.

After editing and trimming vector sequences with GENEIOUS [77], DNA sequences were assessed for gene and sponge identity via BLASTX or BLASTP queries [78], followed by preliminary single-gene phylogenetic analyses under the likelihood framework described below. The identification of likely paralogs followed standard procedures based on the generation of trees including all the members of each gene family that could be identified in GenBank (via reciprocal blasting). Within the context of these trees, paralogy groups were identified and only the sequences nesting within the selected orthology group were used. New sequences generated in this study have been submitted to GenBank (Table 1). Sequences are also available via the Porifera Tree of Life database (PorToL.org). In addition, voucher specimens for many of the sequences presented in Sperling et al. [18], [44] were examined, resulting in several instances of updated taxonomic identification and classification (Table 1).

Nucleotide sequences were translated and aligned using MUSCLE [79] and visualized in SEAVIEW (v. 4.3) [80]. In addition to the new sequences, the initial alignment included data for sponges that had already been published (Table 1). Also, five species for which transcriptome data exist were also added to the dataset. Both mRNA and cDNA from *Corticium candelabrum*, *Petrosia ficiformis* and *Sycon coactum* were obtained using protocols available in Riesgo et al. [81]. *Sycon ciliatum* and *Leucosolenia complicata* sequences are derived from current genome and transcriptome sequencing projects for these species [82] and Adamska, unpublished). *De novo* assemblies of the reads obtained with Illumina GA (Illumina, Solexa, USA) were built with CLC Genomics Workbench 4 (CLCbio, MA, USA). Local blasts against the contig lists generated were used to search for the housekeeping genes. Initially, 50 outgroup taxa representing Bilateria, Ctenophora, Cnidaria, Placozoa and non-metazoan Opisthokonta were included in the analyses. However, preliminary phylogenetic analyses, conducted as described below, indicated that inferred demosponge relationships were robust to outgroup choice and therefore outgroups in the final dataset were reduced to the cnidarian taxa (*Acropora*, *Metridium* and *Nematostella*) and the placozoan *Trichoplax*. Approximately 40 positions in the alignment were manually excluded from analyses because they represented insertions present in one or a small number (<5) of taxa.

### Phylogenetic Analyses

For all gene trees we investigated the presence of significant clustering information using Maximum Likelihood Mapping [83] as implemented in Treepuzzle V. 5.2 [51]. The dataset was analyzed in both Bayesian and Maximum Likelihood (ML) frameworks. For the ML analyses, appropriate models of amino acid evolution were assessed using the Akaike Information Criterion (AIC), as implemented in ProtTest (v.2.4) [49]. The computing cluster of the Smithsonian's Laboratories of Analytical Biology was used to run the parallelized version of RaxML [84] to search for maximum likelihood (ML) topologies. We assumed the model that best fit our data according to the second-order AIC (AICc-1) with the exception that a proportion of invariant sites was not estimated (according to a recommendation in the RaxML manual). We also used RaxML to conduct bootstrap analyses (400 replicate searches) to assess nodal support. We searched for ML topologies using each gene separately as well as all genes combined. We analyzed the combined data a) assuming a single model for all the data and b) by assigning most appropriate models to each gene partition (mixed models).

Bayesian analyses were performed using the site-heterogeneous CAT-GTR+gamma in Phylobayes 3.3b [85]. This model was selected because Bayesian model selection, performed using 10-fold cross-validation [86], showed that CAT-GTR best fitted our dataset, outperforming CAT, GTR and LG. The considered models were: WAG, LG, GTR, CAT, and CAT-GTR (all models used a gamma correction to account for rate heterogeneity among sites). The CAT based models (in this case CAT and CAT-GTR [86]) are mixture models developed to better take into account site-specific features of protein evolution. These models are thus expected to fit the data better than homogeneous time reversible models like LG and GTR [86]. Indeed, CAT based models have previously been shown to fit amino acid datasets better than other models and they have been shown to be highly effective at reducing systematic biases, like long branch attraction, which are well known to be very pervasive in deep time phylogenetics. In Phylobayes two independent analyses were run for 30,000 cycles sampling every 100 points. The analyses were considered converged when the largest discrepancy observed across all bipartitions (i.e. the maxdiff statistics) dropped below 0.15, despite the Phylobayes manual's suggestion that a chain has reached convergence when maxdiff <0.3. Support values for the nodes recovered in the CAT-GTR analysis are expressed as posterior probabilities.

Comparisons were made between the different single-gene topologies and the Bayesian topology to the ML tree derived from the overall data. In addition, nodal differences were calculated, as measured by the root-mean-squared distance, in Topd (v.3.3) [87]. Taxa that were missing data for some genes were pruned from the combined tree prior to calculating nodal differences. Topd was also used to conduct randomization analyses to test whether similarities between the various topologies and the combined ML topology were not greater than expected by chance. Finally, further ML searches were conducted by sequentially excluding the three genes that subtend the trees that are most distant from the tree derived from the concatenated dataset, as measured by subtracting the random nodal difference from the actual nodal difference. To further investigate the extent to which the principal signal [53] in the single-genes corroborated the results of concatenated Bayesian and ML analyses, we performed a supertree analysis. The supertree was built using the Matrix Representation with Parsimony method [53]. Input trees used for this analysis were, for each gene, the 400 bootstrap trees derived (see above) under ML. This set of 2800 input trees was bootstrapped to generate 100 replicate datasets, each of which scored 2800 trees using the software CLANN [88]. For each bootstrapped dataset a bootstrap supertree was recovered and a majority rule consensus of the recovered bootstrap supertrees was built to estimate nodal support.

Finally, analyses were performed to test for tree reconstruction artifacts. More precisely we investigated the potential effect of long-branch attraction and compositional attraction on our results. We first investigated the effect of using alternative model of evolution on our results. We thus built trees (within a Bayesian framework) using models (WAG, LG, GTR, CAT, and CAT-GTR, each with a gamma correction) providing different levels of fit to the data and compared the trees we obtained. We tested whether the taxa in our dataset were compositionally heterogeneous performing a posterior predictive analysis (see for example [18]) of compositional heterogeneity using Phylobayes under the CAT-GTR model. The posterior predictive analysis indicated that several taxa displayed a biased composition of their sites. This, if not addressed, can cause compositional artifacts. To test whether our results were affected by compositional biases we performed two analyses. First we analysed (under CAT-GTR) a dataset from which all compositionally heterogeneous taxa were excluded. This experiment has the downside of excluding potentially important taxa. Accordingly, a second experiment was performed in which our dataset was recoded using the Dayhoff scheme. Dayhoff recoding can alleviate compositional artifact, and a posterior predictive analysis of our Dayhoff-recoded dataset was performed (under CAT-GTR) to evaluate whether further compositionally biased taxa remained after the application of Dayhoff recoding. Finally, our Dayhoff recoded dataset was analysed using both a site-homogeneous (GTR) and a site heterogeneous (CAT-GTR) model.

To test for the potential effect of long-branch attraction artifacts we identified fast evolving sites in our dataset using the program Tiger [89]. After that, sites that Tiger deemed as being fast evolving (bins 7 to 10) were excluded and the slowly evolving sites analysed in isolation. In addition to the site-stripping analysis, we also performed an analysis where all the outgroups to Demospongiae (including Hexactinellida) were removed.

## Supporting Information

Figure S1 Maximum Likelihood Mapping shows ALD has signal to resolve unambiguously over 90% of the quartets that make up the ALD-derived tree. ALD cannot resolve 4.4% of the quartets.
(PDF)

Figure S2 Maximum Likelihood Mapping shows ATPB has signal to resolve unambiguously over 82% of the quartets that make up the ATPB-derived tree. ATPB cannot resolve 8% of the quartets.
(PDF)

Figure S3 Maximum Likelihood Mapping shows CAT has signal to resolve unambiguously over 82% of the quartets that make up the CAT-derived tree. CAT cannot resolve 9% of the quartets.
(PDF)

Figure S4 Maximum Likelihood Mapping shows EF1a has signal to resolve unambiguously over 76% of the quartets that make up the EF1a-derived tree. EF1a cannot resolve 12.3% of the quartets.
(PDF)

Figure S5 Maximum Likelihood Mapping shows MAT has signal to resolve unambiguously nearly 83% of the quartets that make up the MAT-derived tree. MAT cannot resolve 10.2% of the quartets.
(PDF)

Figure S6 Maximum Likelihood Mapping shows PFK has signal to resolve unambiguously over 71% of the quartets that make up the PFKtree. PFK cannot resolve 20.6% of the quartets.
(PDF)

Figure S7 Maximum Likelihood Mapping shows TPI has signal to resolve unambiguously over 76% of the quartets that make up the TPI-derived tree. TPI cannot resolve 15.8% of the quartets.
(PDF)

Figure S8 Maximum Likelihood topology based on ALD, with assumed model of LG+gamma.
(PDF)

Figure S9 Maximum Likelihood topology based on ATPB, with assumed model of WAG+gamma.
(PDF)

Figure S10 Maximum Likelihood topology based on CAT, with assumed model of LG+gamma.
(PDF)

Figure S11 Maximum Likelihood topology based on EF1A, with assumed model of LG+F+gamma.
(PDF)

Figure S12 Maximum Likelihood topology based on MAT, with assumed model of LG+gamma.
(PDF)

Figure S13 Maximum Likelihood topology based on PFK, with assumed model of LG+gamma.
(PDF)

Figure S14 Maximum Likelihood topology based on TPI, with assumed model of LG+gamma.
(PDF)

Figure S15 Maximum Likelihood topology based on NHK6, with assumed model of LG+gamma.
(PDF)

Figure S16 Maximum Likelihood topology based on NHK5, with assumed model of LG+gamma.
(PDF)

Figure S17 Maximum Likelihood topology based on NHK4, with assumed model of LG+gamma.
(PDF)

Figure S18 Consensus supertree derived from the input trees that represents the signal in the collection of the individual trees.
(PDF)

Figure S19 Bayesian analysis of Dayhoff recoded data using CAT-GTR.
(PDF)

Figure S20 Bayesian analysis of Dayhoff recoded data using GTR.
(PDF)

Figure S21 Bayesian analysis using CAT-GTR, with all compositionally heterogenous taxa excluded.
(PDF)

Figure S22 Bayesian analysis using CAT-GTR, excluding fast-evolving sites with Tiger software (“SlowFast Tree”).
(PDF)

Figure S23 Bayesian analysis using CAT-GTR, with no outgroups.
(PDF)

Table S1 Results of the Posterior Predictive Analysis of the combined data set (all 7 genes) under the CAT GTR model. Taxa with a star are heterogeneous in composition.
(PDF)

Table S2 An analysis of the Dayhoff recoded dataset (still under CAT-GTR). As expected, nearly all the heterogeneity is gone (compared to Table S1).
(PDF)

Table S3 Nested primers used to facilitate amplifications of 5 of the 7 genes analyzed in this work.
(PDF)
