# Supplementary material for: Reconstruction of Family-Level Phylogenetic Relationships within Demospongiae (Porifera) Using Nuclear Encoded Housekeeping Genes
Source: PLoS One. 2013 Jan 23;8(1):e50437. doi: 10.1371/journal.pone.0050437 (PMC3553142; doi:10.1371/journal.pone.0050437)

Figure S1. Maximum Likelihood Mapping shows ALD has signal to resolve unambiguously over 90% of the quartets that make up the ALD-derived tree. ALD cannot resolve 4.4% of the quartets.

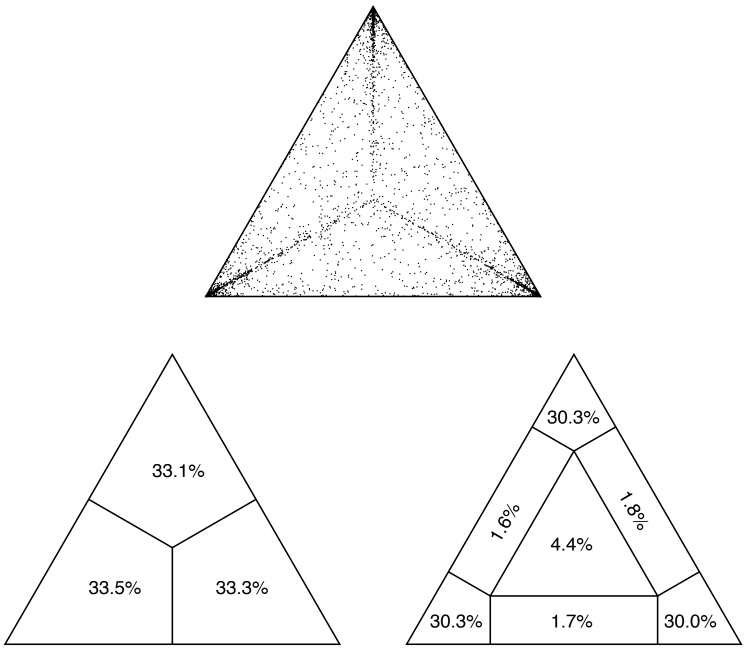

Supplement: Figure S1 — Maximum Likelihood Mapping shows ALD has signal to resolve unambiguously over 90% of the quartets that make up the ALD-derived tree. ALD cannot resolve 4.4% of the quartets. (PDF) [file pone.0050437.s001.pdf]
