# Supplementary material for: Reconstruction of Family-Level Phylogenetic Relationships within Demospongiae (Porifera) Using Nuclear Encoded Housekeeping Genes
Source: PLoS One. 2013 Jan 23;8(1):e50437. doi: 10.1371/journal.pone.0050437 (PMC3553142; doi:10.1371/journal.pone.0050437)

Figure S5. Maximum Likelihood Mapping shows MAT has signal to resolve unambiguously nearly 83% of the quartets that make up the MAT-derived tree.   MAT cannot resolve 10.2% of the quartets.

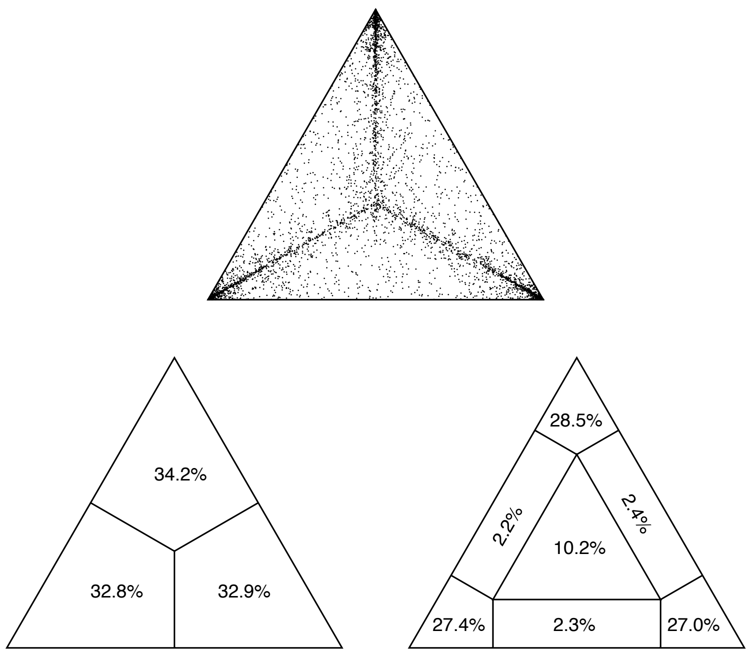

Supplement: Figure S5 — Maximum Likelihood Mapping shows MAT has signal to resolve unambiguously nearly 83% of the quartets that make up the MAT-derived tree. MAT cannot resolve 10.2% of the quartets. (PDF) [file pone.0050437.s005.pdf]
