# Supplementary figures and images for: Reconstruction of Family-Level Phylogenetic Relationships within Demospongiae (Porifera) Using Nuclear Encoded Housekeeping Genes
Source: PLoS One. 2013 Jan 23;8(1):e50437. doi: 10.1371/journal.pone.0050437 (PMC3553142; doi:10.1371/journal.pone.0050437)

Figure S8. Maximum Likelihood topology based on ALD, with assumed model of LG+gamma.

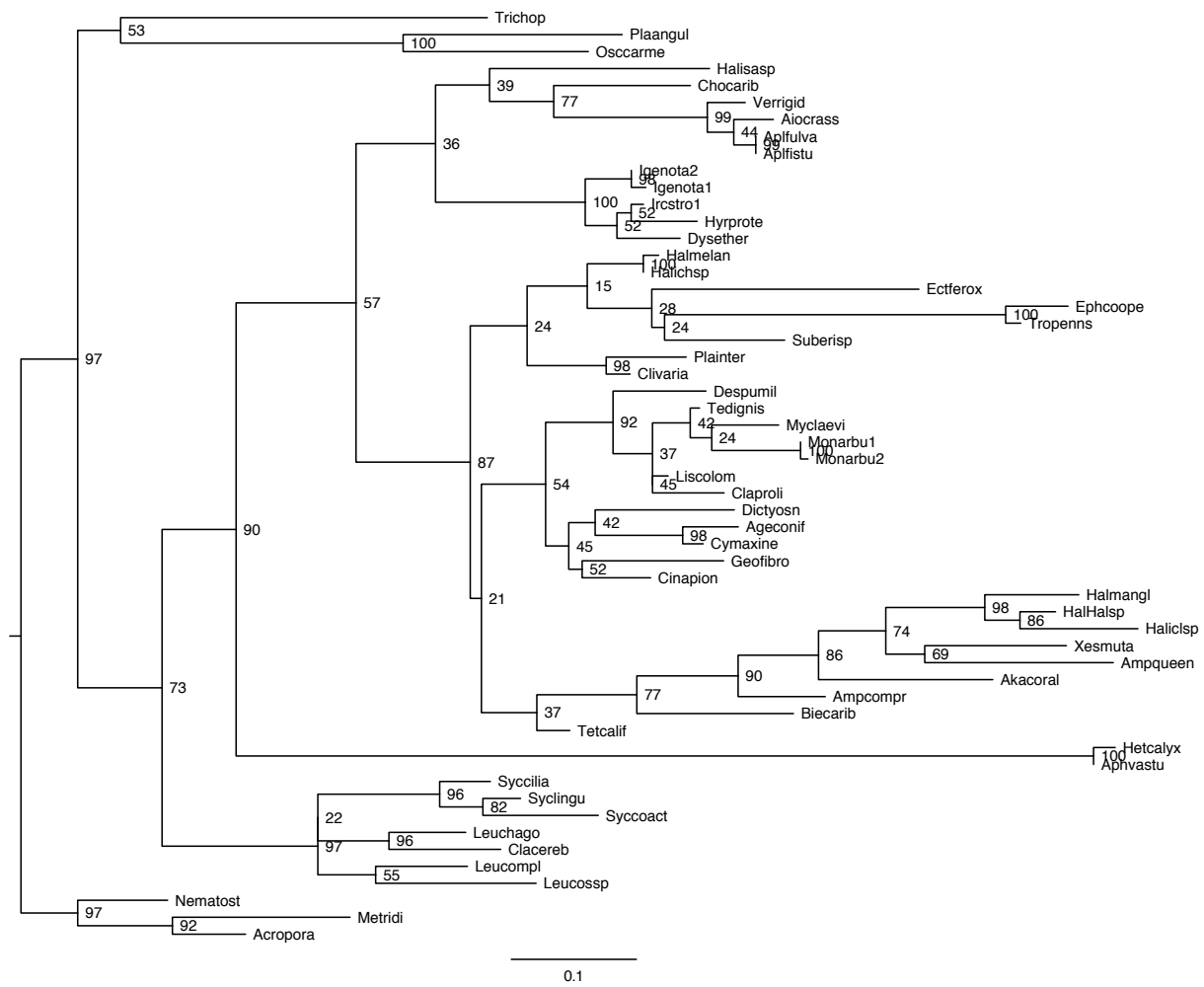

Supplement: Figure S8 — Maximum Likelihood topology based on ALD, with assumed model of LG+gamma. (PDF) [file pone.0050437.s008.pdf]

Figure S9. Maximum Likelihood topology based on ATPB, with assumed model of WAG+gamma.

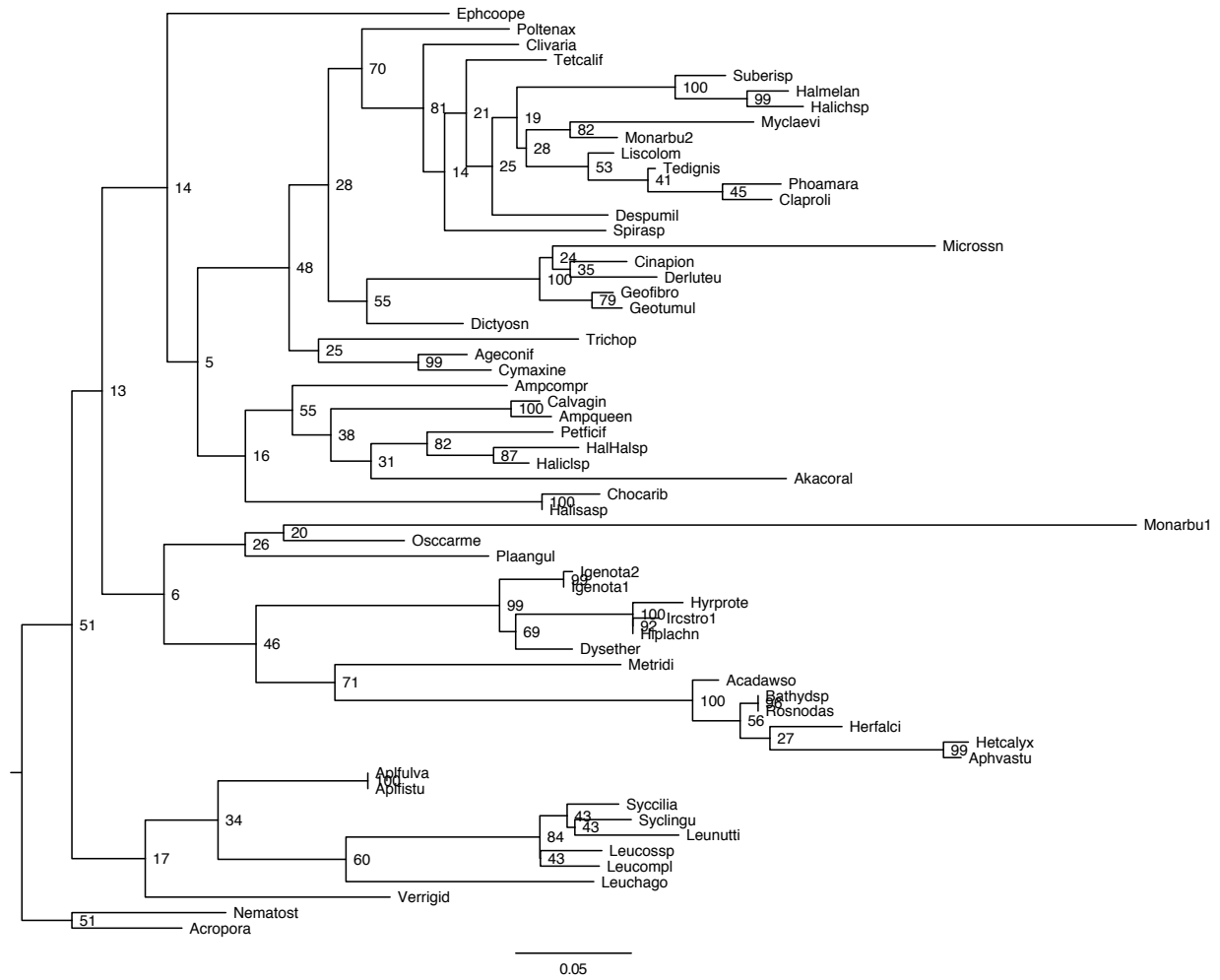

Supplement: Figure S9 — Maximum Likelihood topology based on ATPB, with assumed model of WAG+gamma. (PDF) [file pone.0050437.s009.pdf]

Figure S10. Maximum Likelihood topology based on CAT, with assumed model of LG+gamma.

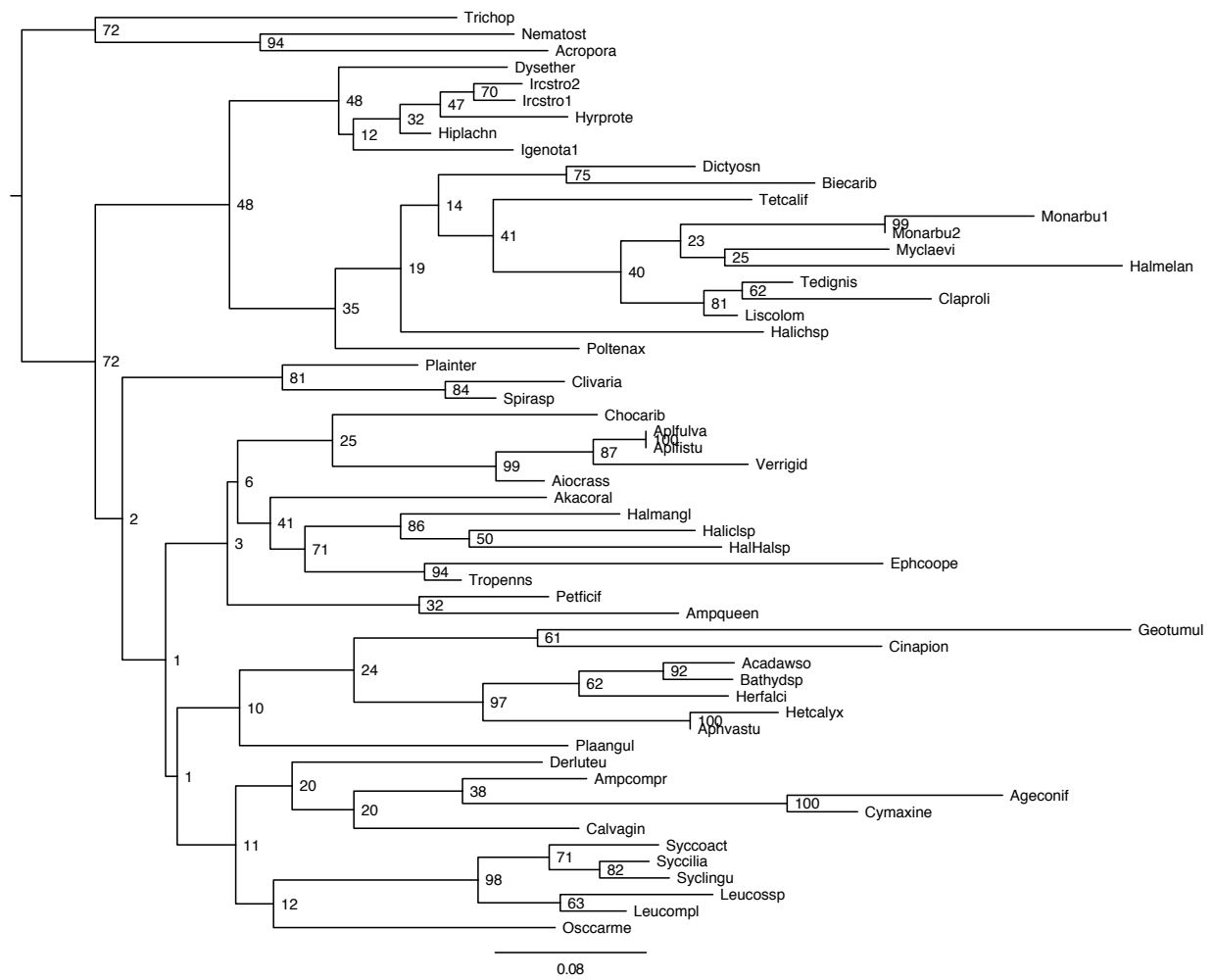

Supplement: Figure S10 — Maximum Likelihood topology based on CAT, with assumed model of LG+gamma. (PDF) [file pone.0050437.s010.pdf]

Figure S11. Maximum Likelihood topology based on EF1A, with assumed model of LG+F+gamma.

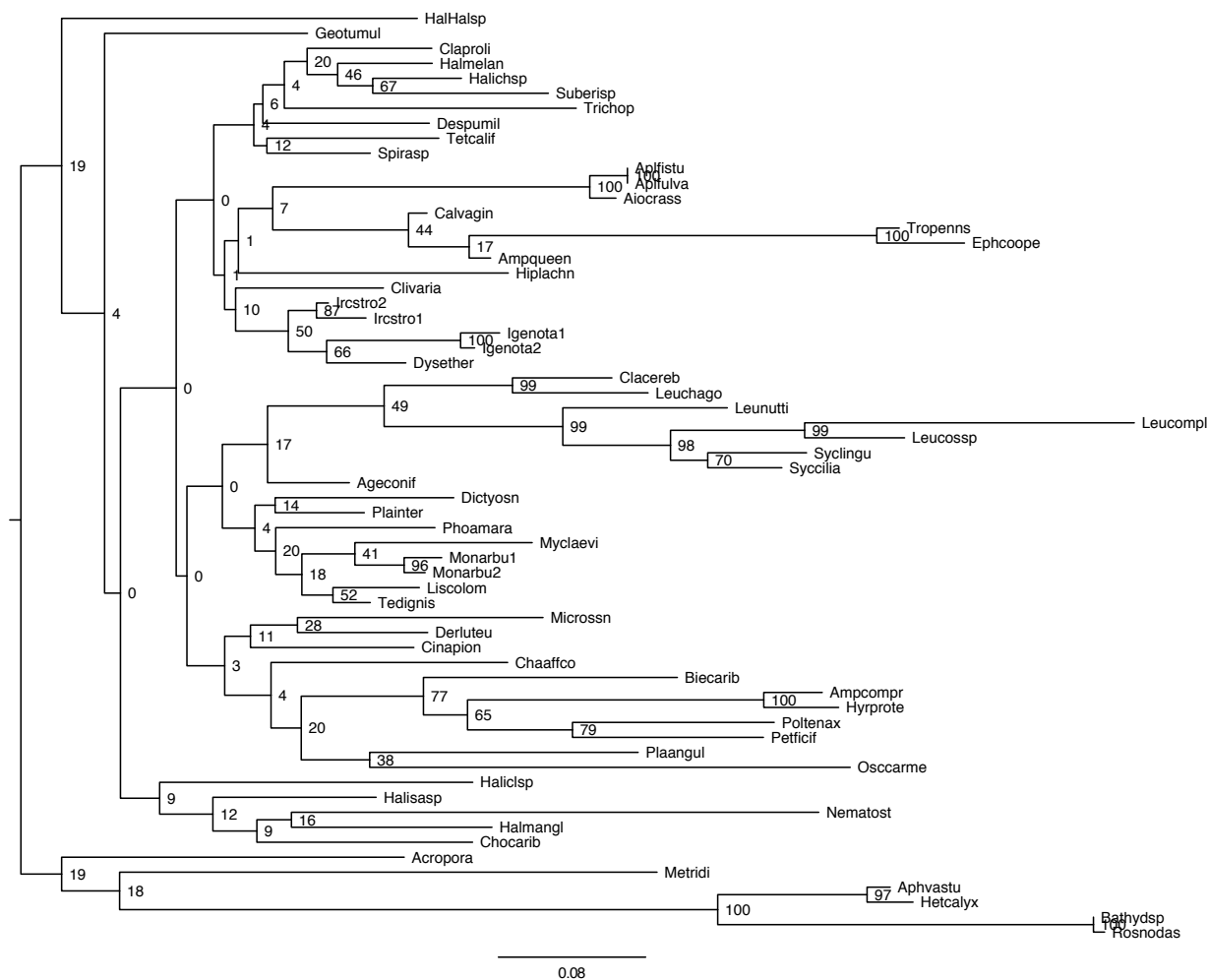

Supplement: Figure S11 — Maximum Likelihood topology based on EF1A, with assumed model of LG+F+gamma. (PDF) [file pone.0050437.s011.pdf]

Figure S12. Maximum Likelihood topology based on MAT, with assumed model of LG+gamma.

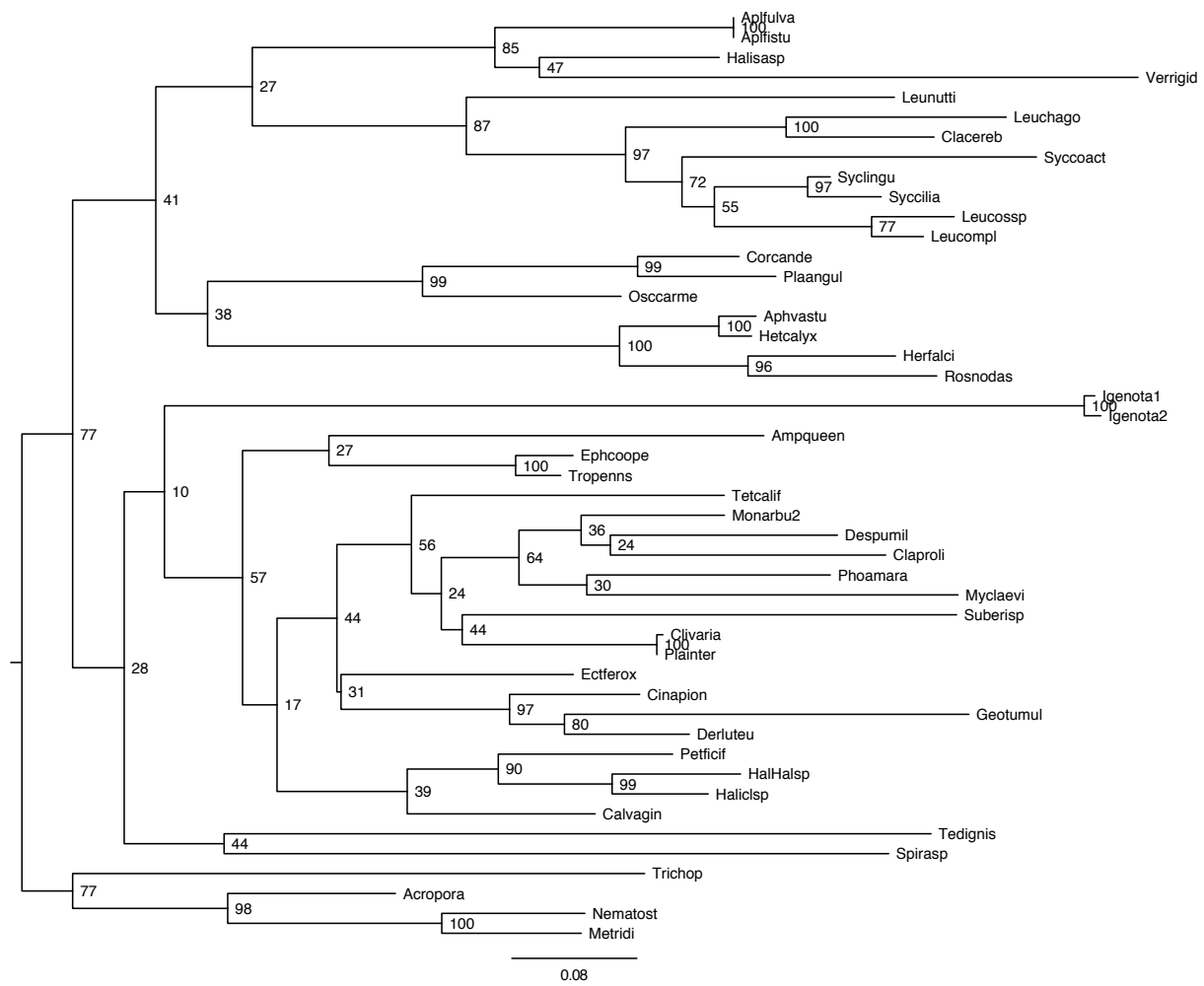

Supplement: Figure S12 — Maximum Likelihood topology based on MAT, with assumed model of LG+gamma. (PDF) [file pone.0050437.s012.pdf]

Figure S13. Maximum Likelihood topology based on PFK, with assumed model of LG+gamma.

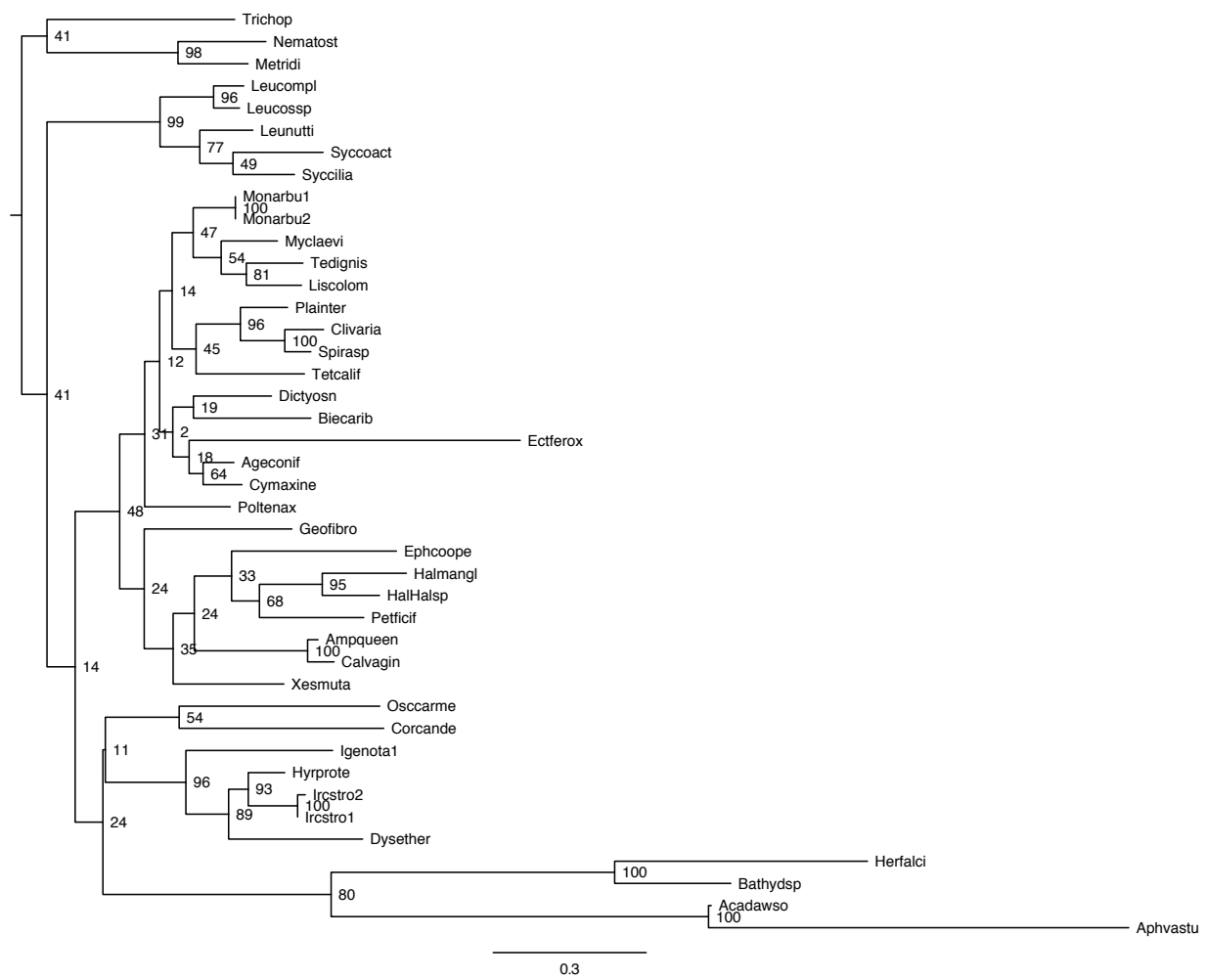

Supplement: Figure S13 — Maximum Likelihood topology based on PFK, with assumed model of LG+gamma. (PDF) [file pone.0050437.s013.pdf]

Figure S14. Maximum Likelihood topology based on TPI, with assumed model of LG+gamma.

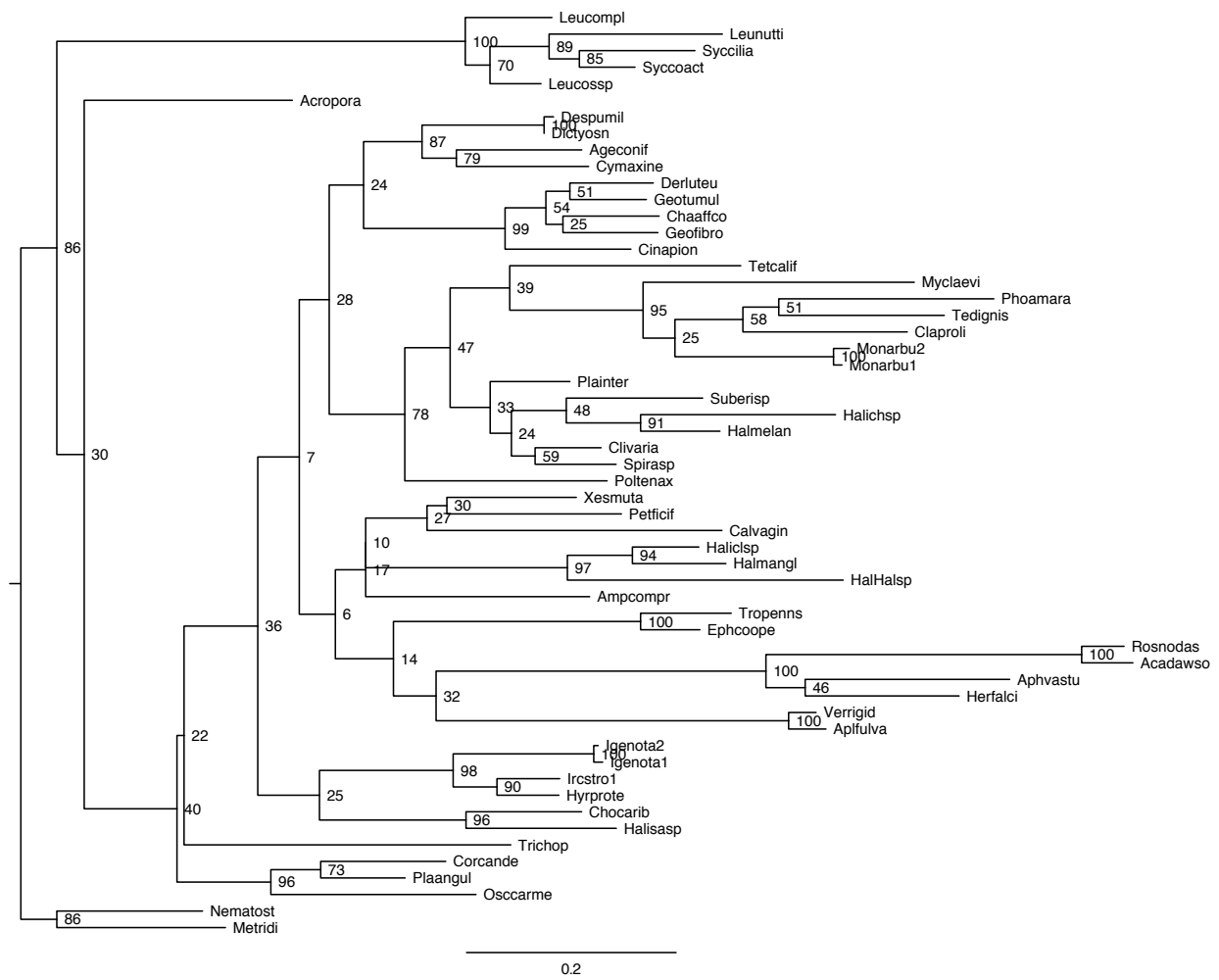

Supplement: Figure S14 — Maximum Likelihood topology based on TPI, with assumed model of LG+gamma. (PDF) [file pone.0050437.s014.pdf]

Figure S15. Maximum Likelihood topology based on NHK6, with assumed model of LG+gamma.

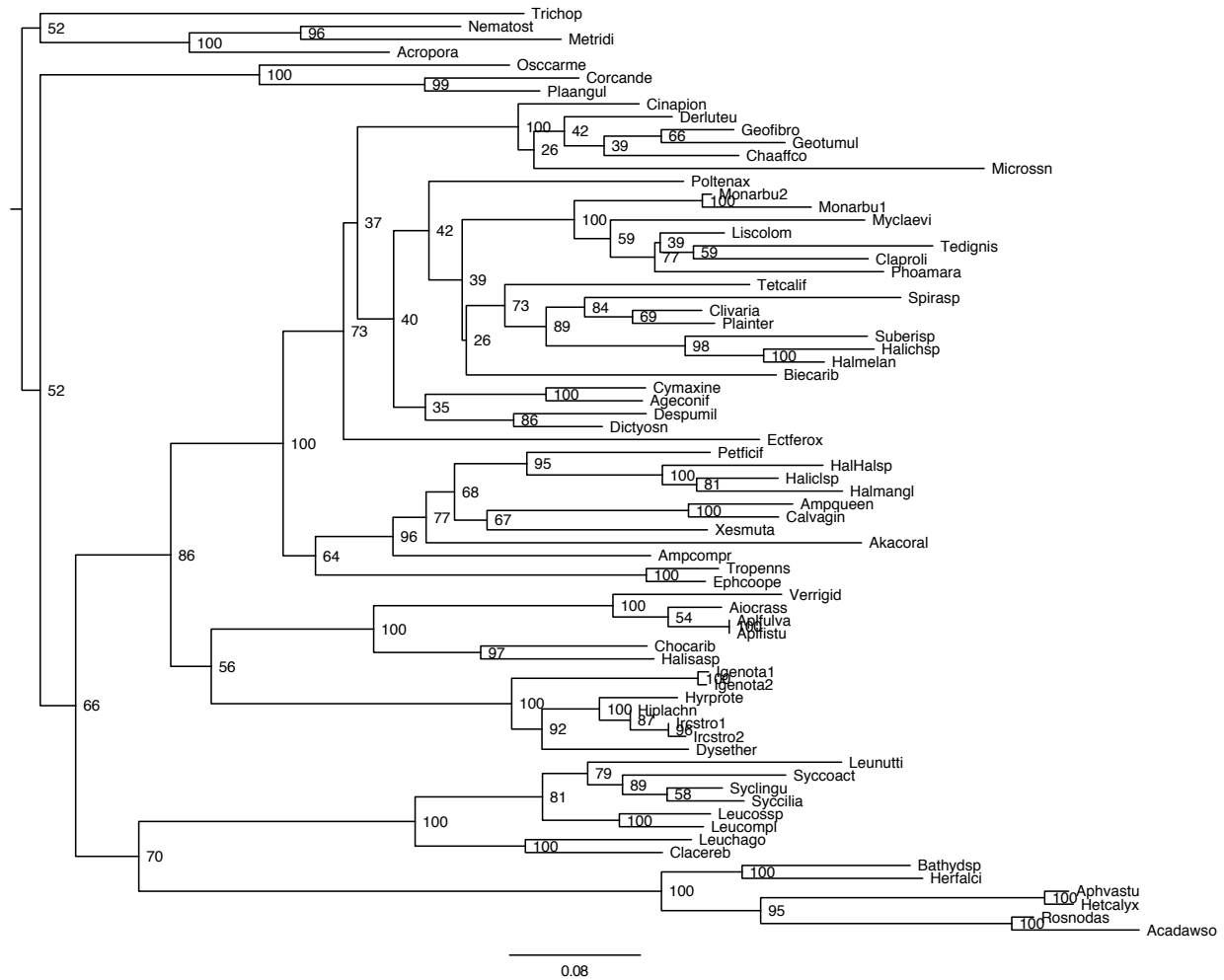

Supplement: Figure S15 — Maximum Likelihood topology based on NHK6, with assumed model of LG+gamma. (PDF) [file pone.0050437.s015.pdf]

Figure S16. Maximum Likelihood topology based on NHK5, with assumed model of LG+gamma.

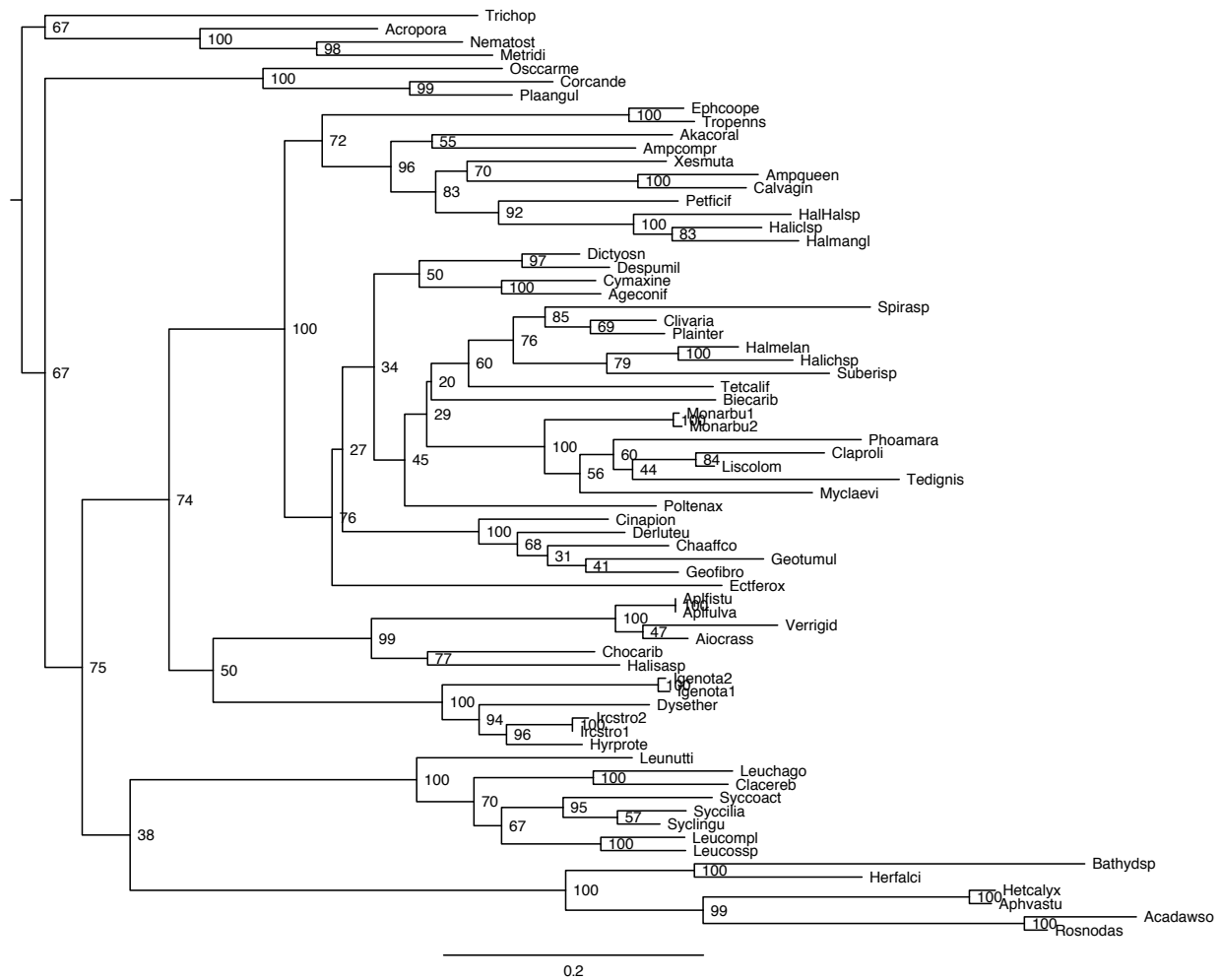

Supplement: Figure S16 — Maximum Likelihood topology based on NHK5, with assumed model of LG+gamma. (PDF) [file pone.0050437.s016.pdf]

Figure S17. Maximum Likelihood topology based on NHK4, with assumed model of LG+gamma.

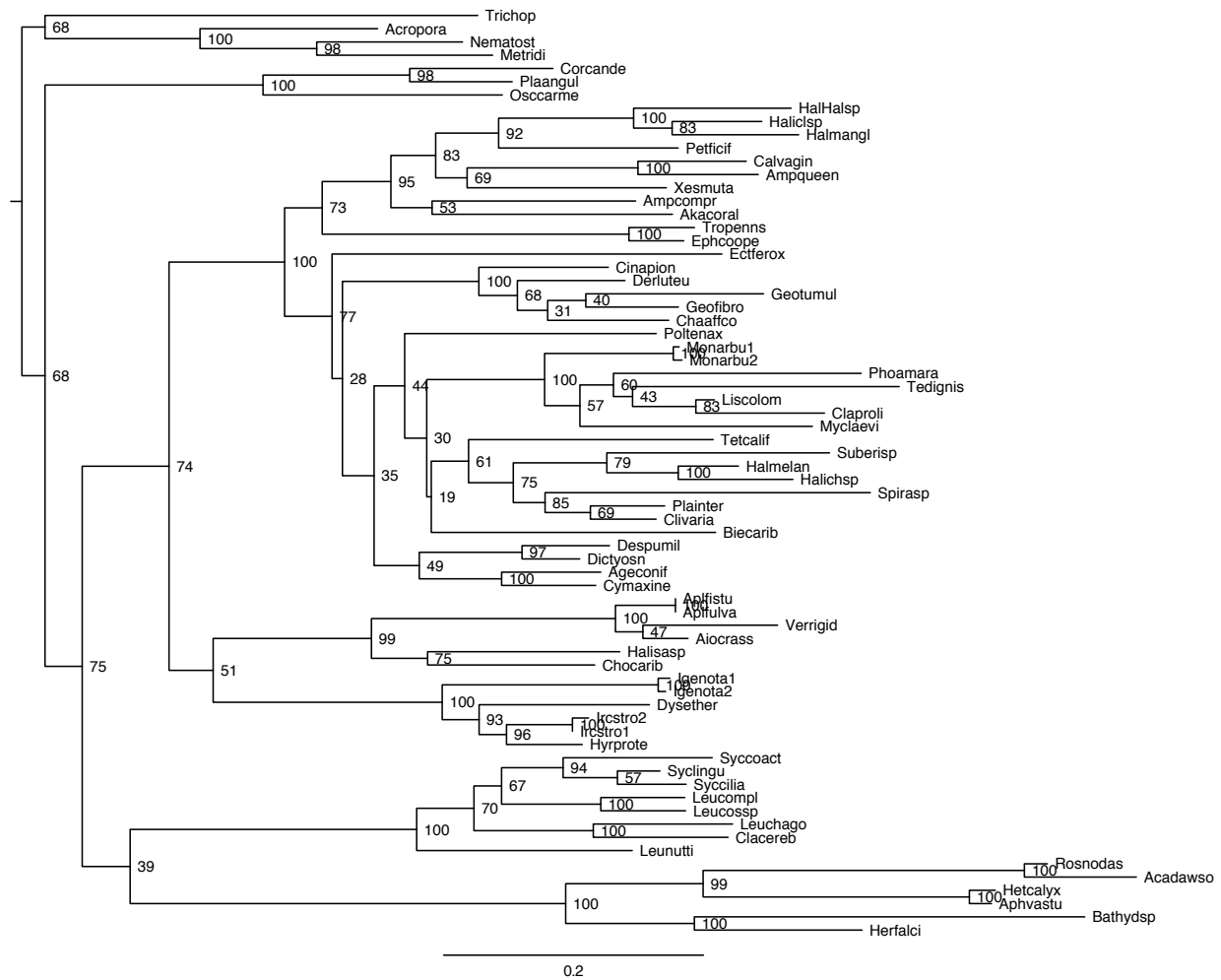

Supplement: Figure S17 — Maximum Likelihood topology based on NHK4, with assumed model of LG+gamma. (PDF) [file pone.0050437.s017.pdf]

Figure S19. Bayesian analysis of Dayhoff recoded data using CAT-GTR.

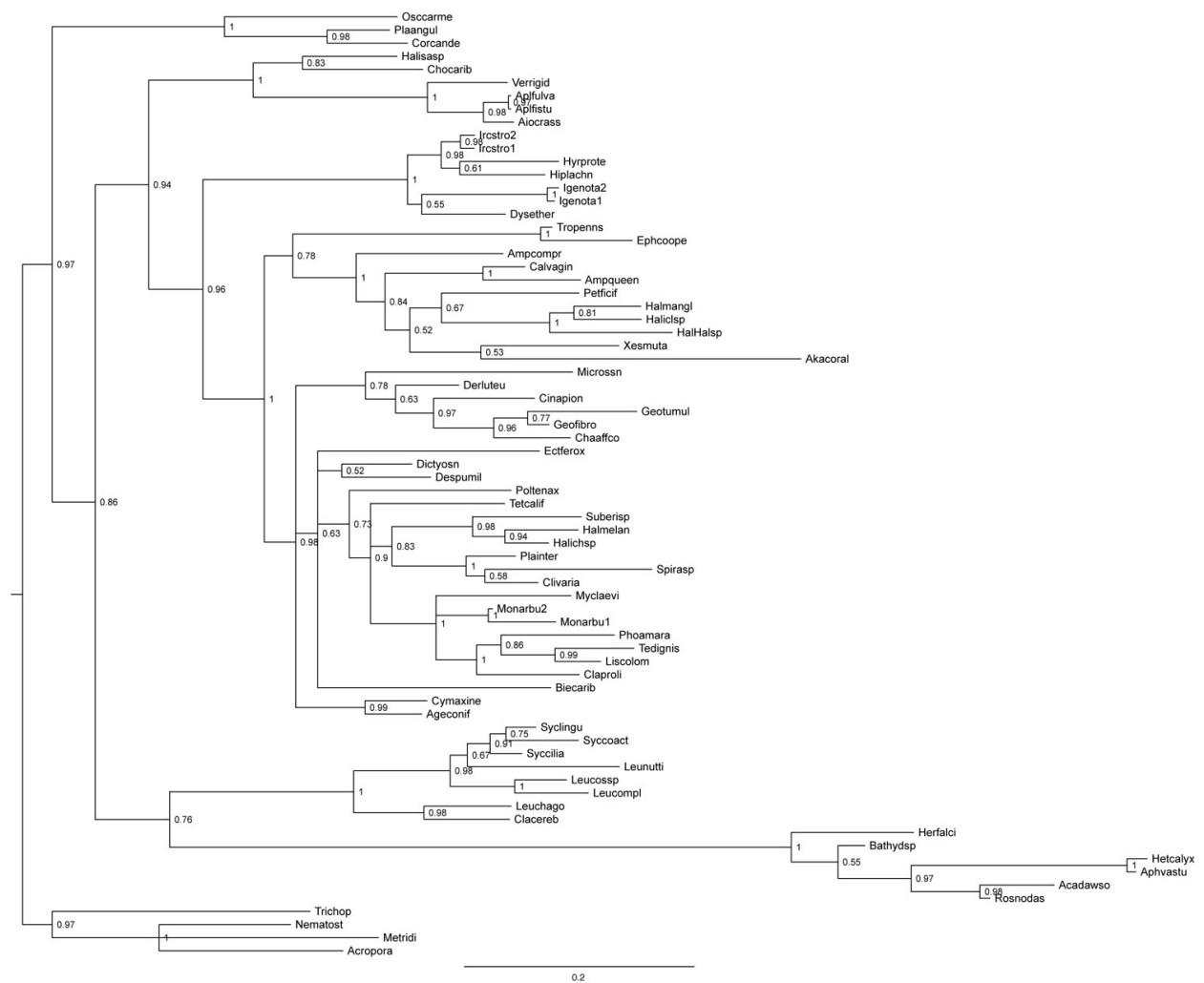

Supplement: Figure S19 — Bayesian analysis of Dayhoff recoded data using CAT-GTR. (PDF) [file pone.0050437.s019.pdf]

Figure S20. Bayesian analysis of Dayhoff recoded data using GTR.

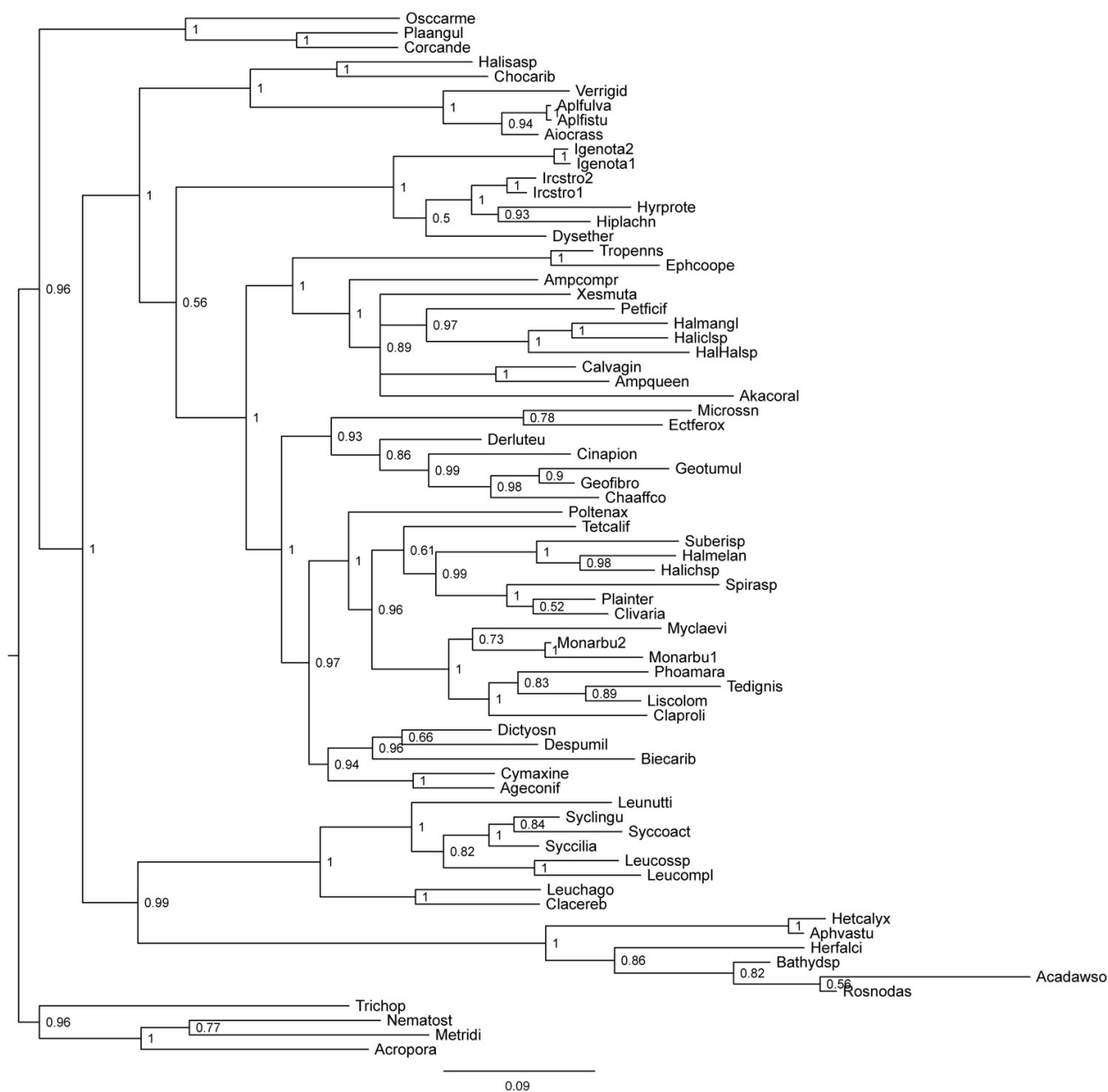

Supplement: Figure S20 — Bayesian analysis of Dayhoff recoded data using GTR. (PDF) [file pone.0050437.s020.pdf]

Figure S23. Bayesian analysis using CAT-GTR, with no outgroups.

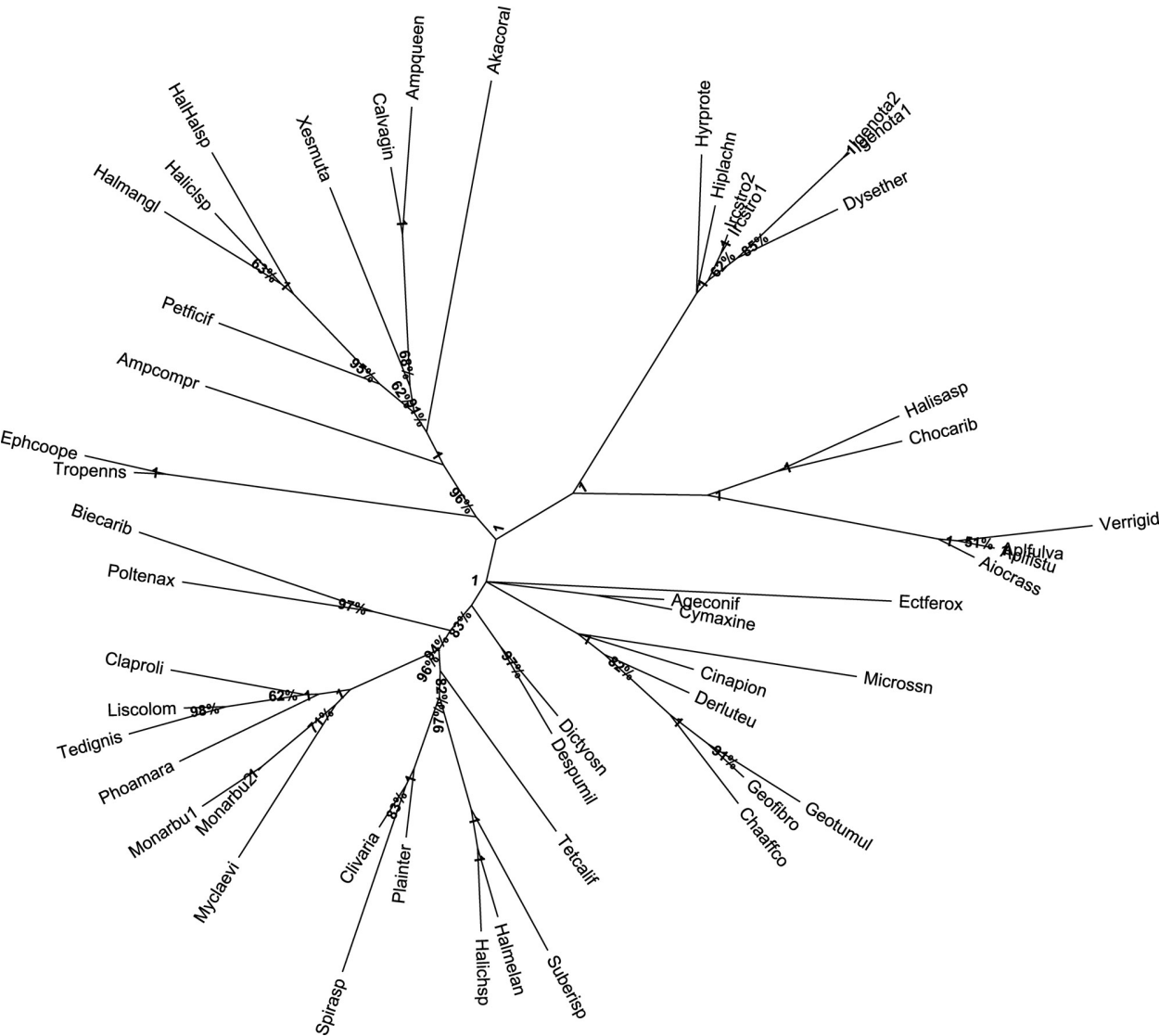

Supplement: Figure S23 — Bayesian analysis using CAT-GTR, with no outgroups. (PDF) [file pone.0050437.s023.pdf]
