# Supplementary material for: Reconstruction of Family-Level Phylogenetic Relationships within Demospongiae (Porifera) Using Nuclear Encoded Housekeeping Genes
Source: PLoS One. 2013 Jan 23;8(1):e50437. doi: 10.1371/journal.pone.0050437 (PMC3553142; doi:10.1371/journal.pone.0050437)

Figure S21. Bayesian analysis using CAT-GTR, with all compositionally heterogenous taxa excluded.

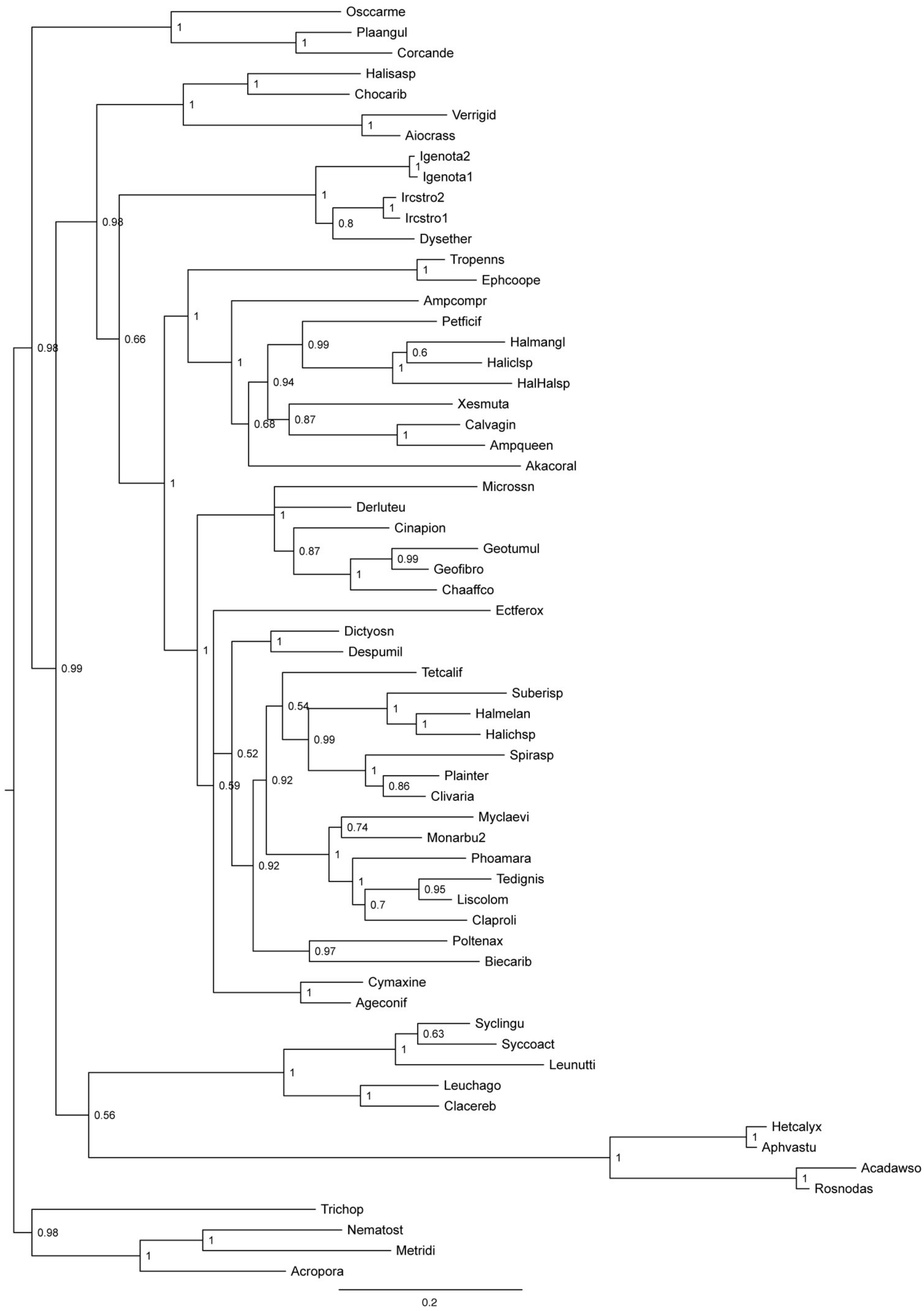

Supplement: Figure S21 — Bayesian analysis using CAT-GTR, with all compositionally heterogenous taxa excluded. (PDF) [file pone.0050437.s021.pdf]
