# Supplementary material for: Reconstruction of Family-Level Phylogenetic Relationships within Demospongiae (Porifera) Using Nuclear Encoded Housekeeping Genes
Source: PLoS One. 2013 Jan 23;8(1):e50437. doi: 10.1371/journal.pone.0050437 (PMC3553142; doi:10.1371/journal.pone.0050437)

Figure S22. Bayesian analysis using CAT-GTR, excluding fast-evolving sites with Tiger software (“SlowFast Tree”).

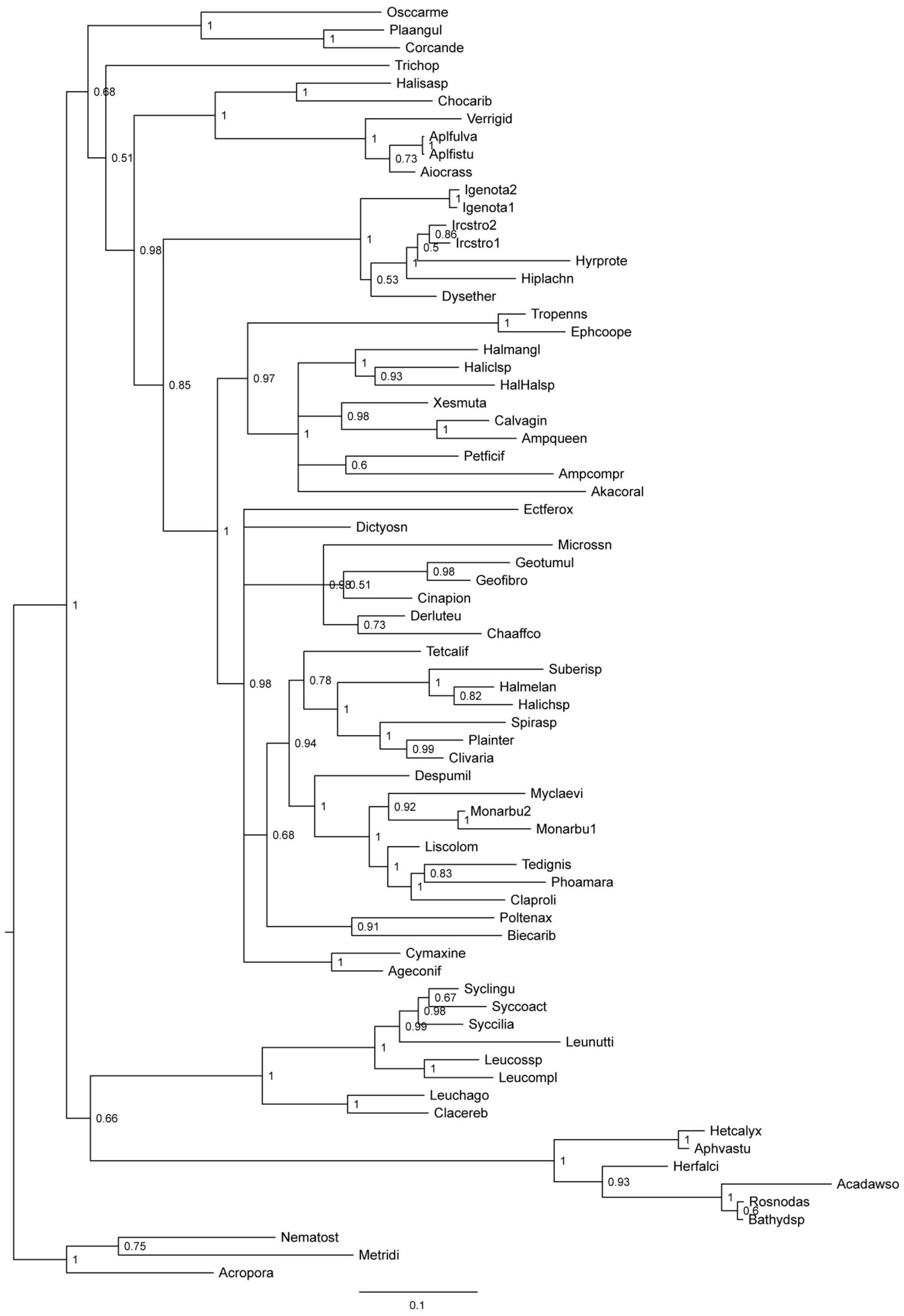

Supplement: Figure S22 — Bayesian analysis using CAT-GTR, excluding fast-evolving sites with Tiger software (“SlowFast Tree”). (PDF) [file pone.0050437.s022.pdf]
