# Supplementary material for: Reconstruction of Family-Level Phylogenetic Relationships within Demospongiae (Porifera) Using Nuclear Encoded Housekeeping Genes
Source: PLoS One. 2013 Jan 23;8(1):e50437. doi: 10.1371/journal.pone.0050437 (PMC3553142; doi:10.1371/journal.pone.0050437)
Supplement: Table S1 — Results of the Posterior Predictive Analysis of the combined data set (all 7 genes) under the CAT GTR model. Taxa with a star are heterogeneous in composition. (PDF) [file pone.0050437.s024.pdf]

Table S1. Results of the Posterior Predictive Analysis of the combined data set (all 7 genes) under the CAT GTR model. Taxa with a star are heterogeneous in composition.

	taxon	p-value	z-score
	Nematost	0.791	-0.88
	Metridi	0.97	-1.432
	Acropora	0.626	-0.494
	Trichop	0.985	-1.428
	Spirasp	0.761	-0.835
	Ageconif	0.089	1.288
	Geofibro	0.104	1.286
	Aiocrass	0.686	-0.62
*	Aplfistu	0.044	1.761
*	Aplfulva	0	3.46
	Verrigid	0.149	0.975
	Cymaxine	0.059	1.722
	Despumil	0.85	-0.965
	Dictyosn	0.104	1.358
	Microssn	0.805	-0.766
	Calvagin	0.805	-0.869
	Derluteu	0.641	-0.511
	Haliclsp	0.358	0.436
	HalHalsp	0.641	-0.457
	Halmangl	0.671	-0.532
	Chocarib	0.97	-1.844
	Clivaria	0.88	-1.115
	Liscolom	0.208	0.905
	Monarbu2	0.238	0.868
	Igenota1	0.059	1.627
	Biecarib	0.88	-1.066
	Igenota2	0.238	0.656
	Dysether	0.492	0.014
	Geotumul	0.283	0.475
	Halichsp	0.656	-0.498
	Halmelan	0.059	1.6
	Halisasp	0.641	-0.408
	Phoamara	0.298	0.314
	Ircstro1	0.462	-0.063
	Ircstro2	0.686	-0.454
	Claproli	0.253	0.531
	Myclaevi	0.223	0.582
	Ampqueen	0.865	-1.062
	Ampcompr	0.91	-1.173
	Chaaffco	0.238	0.534
	Xesmuta	0.731	-0.71
	Petficif	0.701	-0.448
	Akacoral	0.402	0.257
	Plainter	0.731	-0.771
	Poltenax	0.731	-0.551

	Ectferox	0.373	0.56
*	Monarbu1	0	2.989
*	Hiplachn	0.014	2.206
	Tropenns	0.119	1.315
	Ephcoope	1	-1.878
	Suberisp	0.85	-0.977
	Tedignis	0.104	1.01
	Tetcalif	0.597	-0.395
	Cinapion	0.925	-1.272
*	Hyrprote	0.029	1.79
	Osccarme	0.701	-0.597
	Corcande	0.91	-1.2
	Plaangul	0.701	-0.557
	Clacereb	0.552	-0.008
	Leuchago	0.447	0.003
	Leunutti	0.089	1.366
*	Leucossp	0	5.807
*	Leucompl	0	6.756
	Syccoact	0.149	1.233
	Syclingu	0.686	-0.505
*	Syccilia	0	2.44
	Hetcalyx	0.164	0.948
	Aphvastu	0.686	-0.578
	Acadawso	0.492	-0.075
	Rosnodas	0.402	0.192
*	Bathydsp	0	2.675
*	Herfalci	0	4.126

```
global test: succeeded
observed   : 0.0018469
mean pred  : 0.00215426
p-value    : 0.776119
z-score    : -0.844036
```
