# Supplementary material for: Reconstruction of Family-Level Phylogenetic Relationships within Demospongiae (Porifera) Using Nuclear Encoded Housekeeping Genes
Source: PLoS One. 2013 Jan 23;8(1):e50437. doi: 10.1371/journal.pone.0050437 (PMC3553142; doi:10.1371/journal.pone.0050437)
Supplement: Table S2 — An analysis of the Dayhoff recoded dataset (still under CAT-GTR). As expected, nearly all the heterogeneity is gone (compared to Table S1). (PDF) [file pone.0050437.s025.pdf]

Table S2. An analysis of the Dayhoff recoded dataset (still under CAT-GTR). As expected, nearly all the heterogeneity is gone (compared to Table S1).

taxon	p-value	z-score
Nematost	1	-1.214
Metridi	0.881	-0.99
Acropora	0.655	-0.586
Trichop	0.559	-0.324
Spirasp	0.623	-0.452
Ageconif	0.709	-0.624
Geofibro	0.967	-1.299
Aiocrass	0.817	-0.812
Aplfistu	0.365	0.056
Aplfulva	0.494	-0.355
Verrigid	0.44	-0.137
Cymaxine	0.29	0.486
Despumil	0.204	0.66
Dictyosn	0.419	-0.082
Microssn	0.483	-0.204
Calvagin	0.763	-0.761
Derluteu	0.774	-0.752
Haliclsp	0.354	0.246
HalHalsp	0.935	-0.877
Halmangl	0.72	-0.739
Chocarib	0.86	-0.93
Clivaria	0.741	-0.7
Liscolom	0.526	-0.451
Monarbu2	0.978	-1.068
Igenota1	0.677	-0.673
Biecarib	0.763	-0.707
Igenota2	0.849	-0.965
Dysether	0.483	-0.201
Geotumul	0.075	1.805
Halichsp	0.935	-0.994
Halmelan	0.139	1.275
Halisasp	0.44	-0.173
Phoamara	0.86	-0.967
Ircstro1	0.655	-0.566
Ircstro2	0.322	0.182
Claproli	0.752	-0.766
Myclaevi	0.795	-0.772
Ampqueen	0.193	0.556
Ampcompr	0.462	-0.053
Chaaffco	0.129	1.343
Xesmuta	0.967	-1.315
Petficif	0.677	-0.508
Akacoral	0.096	1.208
Plainter	0.462	-0.21
Poltenax	0.258	0.393

	Ectferox	0.623	-0.469
	Monarbul	0.086	1.594
	Hiplachn	0.43	0.096
	Tropenns	0.215	0.76
	Ephcoope	0.763	-0.785
	Suberisp	0.118	1.087
	Tedignis	0.935	-0.993
	Tetcalif	0.075	1.601
	Cinapion	0.526	-0.277
	Hyrprote	0.322	0.021
	Osccarme	0.838	-0.965
	Corcande	0.892	-1.11
	Plaangul	0.87	-0.937
	Clacereb	0.279	0.541
	Leuchago	0.752	-0.683
	Leunutti	0.129	0.997
	Leucossp	0.193	0.74
	Leucompl	0.086	1.239
	Syccoact	0.774	-0.768
	Syclingu	0.967	-1.312
	Syccilia	0.795	-0.744
	Hetcalyx	0.322	0.419
	Aphvastu	0.139	1.117
	Acadawso	0.053	1.829
	Rosnodas	0.193	0.708
*	Bathydsp	0.032	2.097
	Herfalci	0.279	0.15

```
global test: succeeded
observed   : 0.00397486
mean pred  : 0.00395948
p-value    : 0.419355
z-score    : 0.0112307
```
