# Supplementary material for: Reconstruction of Family-Level Phylogenetic Relationships within Demospongiae (Porifera) Using Nuclear Encoded Housekeeping Genes
Source: PLoS One. 2013 Jan 23;8(1):e50437. doi: 10.1371/journal.pone.0050437 (PMC3553142; doi:10.1371/journal.pone.0050437)
Supplement: Table S3 — Nested primers used to facilitate amplifications of 5 of the 7 genes analyzed in this work. (PDF) [file pone.0050437.s026.pdf]

Table S3. Nested primers used to facilitate amplifications of 5 of the 7 genes analyzed in this work.

**Housekeeping Nested Primers**	**Sequence**
ALD nested forward	5’-GAGAGCACDGGCACHATBGG-3’
ALD nested reverse	5’-CTTMARVACACACCTCCAYTTRGC-3’
ATPB nested forward	5’-ATHGCNATGGAYGGNACNGARGG-3’
CAT nested forward	5’-GGNTAYTTYGARGTNACNWSNCA-3’
MAT nested forward	5’-GCVACVAAGACYGGWATGGT-3’
MAT nested reverse	5’-GAGAAGGCYCCRCCYCCATG-3’
TPI nested forward	5’-GCNTAYGARCCNGTNTGGGC-3’
TPI nested reverse	5’-GCCCANACNGGYTCRTANGC-3’
